# A CAF-Associated Stromal Remodeling Signature Links Immune Exclusion to Exhaustion-Prone CD8^+^ T-Cell Dysfunction in High-Grade Serous Ovarian Cancer

**DOI:** 10.3390/ijms27136092

**Published:** 2026-07-07

**Authors:** Yang Bai, Ruifang Chen, Xin Lu

**Affiliations:** 1 Obstetrics and Gynecology Hospital, Fudan University, Shanghai 200011, China; 20111250022@fudan.edu.cn (Y.B.); fc1570@fudan.edu.cn (R.C.); 2 Shanghai Key Laboratory of Reproduction and Development, Shanghai 200433, China; 3 Shanghai Key Laboratory of Female Reproductive Endocrine Related Diseases, Shanghai 200433, China

**Keywords:** high-grade serous ovarian carcinoma, cancer-associated fibroblasts, extracellular matrix remodeling, immune exclusion, T-cell exhaustion

## Abstract

High-grade serous ovarian carcinoma (HGSOC) shows limited benefit from immune checkpoint blockade, partly because stromal barriers impair antitumor immunity. We developed a cancer-associated fibroblast (CAF)-associated mitochondrial metabolic and matrix-remodeling signature, termed CMMS, to characterize this immune-suppressive stromal state. CMMS integrated contractile/myCAF, extracellular matrix (ECM), and mitochondrial metabolic genes. Its clinical, metabolic, and immune relevance was evaluated in TCGA-HGSOC, independent GEO cohorts, single-cell RNA-seq datasets, and an anti-PD-L1-treated cohort, followed by cell–cell communication and experimental validation. LASSO-weighted CMMS stratified overall survival, with high CMMS indicating poorer prognosis. CMMS-high tumors exhibited ECM/TGFβ activation; associations with COL1A1, POSTN, and LOX; and a hypoxia-dominant metabolic phenotype. Mediation analysis suggested that hypoxia largely linked CMMS to glycolytic remodeling. Immune profiling revealed stromal-rich immune exclusion, checkpoint activation, and exhaustion-prone T-cell dysfunction. Single-cell analysis localized CMMS mainly to myCAF-like ECM-remodeling CAFs. In validation datasets, CMMS-high CAFs were associated with reduced CD8 abundance, increased CD8 exhaustion, and stronger matrix- and chemokine-related communication with T cells. Experiments further supported a link between TGFβ-related fibroblast activation, ECM-remodeling features, and impaired CD8^+^ T-cell effector function. Overall, CMMS defines a CAF-enriched fibrotic–hypoxic stromal program associated with immune exclusion-related features, exhaustion-prone T-cell dysfunction, and poor outcome in HGSOC.

## 1. Introduction

High-grade serous ovarian carcinoma (HGSOC) remains the most lethal subtype of ovarian cancer, largely owing to late diagnosis, extensive intraperitoneal dissemination, and frequent relapse after platinum-based chemotherapy [1]. Although immune checkpoint blockade has reshaped the treatment landscape of several solid tumors, its clinical benefit in ovarian cancer has been modest and inconsistent [2,3,4]. This limited efficacy suggests that the ovarian tumor microenvironment contains dominant non-T-cell-intrinsic barriers that restrict effective antitumor immunity [4,5].

As a dominant stromal cell population in HGSOC, CAFs orchestrate microenvironmental remodeling by coordinating extracellular matrix organization, myofibroblastic activation, paracrine signaling, and metabolic adaptation [6]. In particular, myofibroblastic CAF and collagen-rich matrix-remodeling CAF states can generate a dense fibrotic stroma, which not only increases tissue stiffness but also alters nutrient and oxygen availability [7,8]. Such stromal remodeling may establish a hypoxic and glycolytic niche, thereby shaping immune cell localization and function [9,10]. However, how CAF-associated matrix remodeling and metabolic stress are integrated into immune exclusion and T-cell exhaustion in HGSOC remains incompletely defined.

Immune evasion in ovarian cancer is not restricted to the absence of cytotoxic lymphocytes. Tumors may occupy different immune states, from immune-desert lesions to stromal-excluded tumors and immune-infiltrated tumors with exhaustion-prone T-cell dysfunction [11,12]. Immune-excluded tumors are characterized by CD8^+^ T cells that remain spatially confined to stromal regions, with limited access to tumor nests. Conversely, in immune-infiltrated tumors, chronic antigen exposure, hypoxia, lactate accumulation, and immunosuppressive stromal signaling may promote exhaustion-related transcriptional programs [13,14]. Hypoxia can reinforce T-cell exhaustion by inducing mitochondrial stress under chronic stimulation [13]. Hypoxia- and glycolysis-induced lactic acid metabolism impairs T/NK-cell activation and tumor immune surveillance [15]. Thus, a stromal program capable of both restricting T-cell entry and impairing T-cell function could represent a key barrier to effective antitumor immunity.

Mitochondrial and metabolic adaptations in CAFs represent an emerging stromal feature that may support persistent fibroblast activation and matrix remodeling [16,17,18]. CAFs and activated fibroblasts can undergo mitochondrial and bioenergetic remodeling in response to TGFβ signaling, hypoxia, and mechanical cues from the extracellular matrix, thereby supporting myofibroblastic activation and persistent ECM remodeling [19,20]. Nevertheless, most existing stromal signatures focus primarily on fibroblast abundance or ECM genes, without capturing the combined contribution of contractile activation, matrix remodeling, and mitochondrial metabolic adaptation. A composite signature integrating these features may better reflect the functional stromal state that drives immune remodeling in HGSOC.

Here, we sought to determine whether an integrated CAF-associated stromal-metabolic signature could identify the fibrotic and immune-suppressive microenvironmental state underlying poor prognosis and immunotherapy resistance in HGSOC. In this study, we developed a CAF-associated stromal remodeling signature, termed CMMS, by integrating genes related to contractile/myCAF activation, ECM-remodeling, and fibroblast mitochondrial/metabolic features. Across TCGA-HGSOC and independent GEO cohorts, we explored whether CMMS delineates a poor-prognosis stromal state characterized by fibrotic remodeling, hypoxic–glycolytic stress, immune exclusion, and exhaustion-prone T-cell dysfunction. Single-cell transcriptomic analyses further defined the cellular origin of CMMS and resolved CMMS-high CAF states at single-cell resolution. Together, CMMS captures a CAF–ECM-remodeling-driven fibro-hypoxic-glycolytic program. This program primarily shapes immune exclusion through TGFβ-associated stromal barriers, while in the subset of T-cell-inflamed tumors, CMMS is also linked to T-cell phenotypes that exhibit heightened exhaustion, together contributing to immunotherapy resistance and poor prognosis.

## 2. Results

### 2.1. CMMS Is Associated with Poor Prognosis and Fibrotic Stromal Remodeling in HGSOC

**Figure 1:**
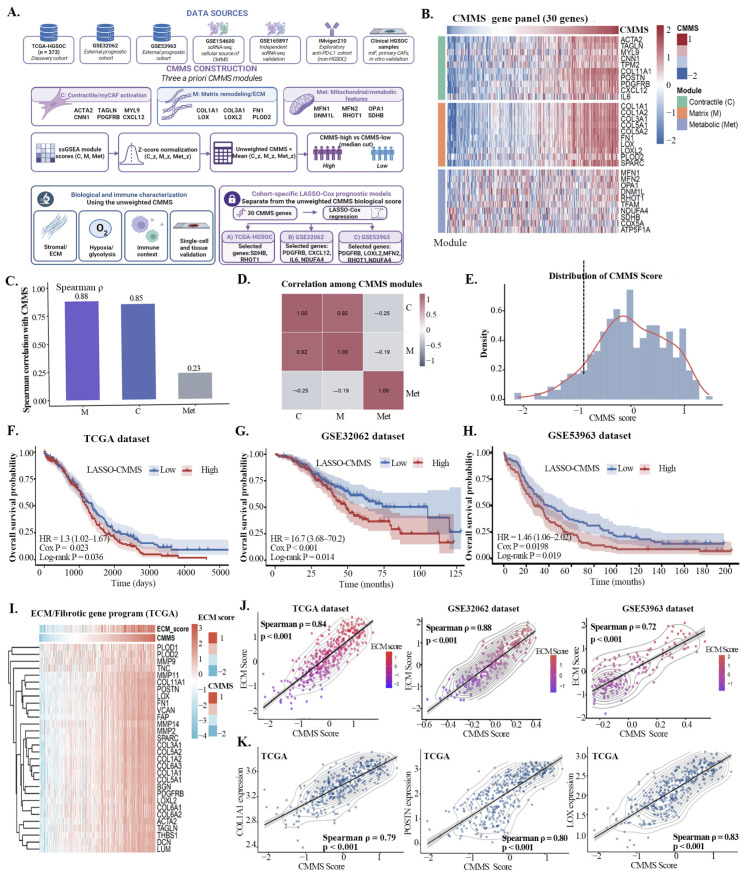
CMMS is associated with poor prognosis and fibrotic stromal remodeling in HGSOC. (**A**) Schematic overview of CMMS construction and analytical workflow (representative genes are shown; the complete gene list is provided in Supplementary Table S3). (**B**) Heatmap showing the expression pattern of the 30 CMMS genes in TCGA-HGSOC samples ranked by CMMS score. (**C**) Spearman correlation between the composite CMMS score and each individual module score in TCGA-HGSOC samples. (**D**) Heatmap showing pairwise Spearman correlations among the contractile/myCAF, matrix remodeling/ECM, and mitochondrial metabolic modules. (**E**) Density distribution of CMMS scores across TCGA-HGSOC samples. (**F**) Kaplan–Meier survival plot for TCGA HGSOC patients stratified by LASSO-weighted CMMS risk score. (**G**) Kaplan–Meier survival analysis of patients in the GSE32062 cohort stratified by the LASSO-weighted CMMS risk score. (**H**) Kaplan–Meier survival analysis in the GSE53963 cohort stratified by the LASSO-weighted CMMS risk score. Hazard ratios were calculated from Cox proportional hazards models, and between-group survival differences were evaluated using the log-rank test. (**I**) Heatmap of ECM/fibrotic gene expression in the TCGA cohort, annotated with patient-level ECM score and CMMS score. (**J**) Density scatter plots showing positive Spearman correlations between CMMS score and ECM score in TCGA, GSE32062, and GSE53963 datasets. (**K**) Density scatter plots demonstrating significant positive correlations between CMMS score and the expression of COL1A1, POSTN, and LOX in the TCGA ovarian cancer dataset.

To characterize CAF-associated stromal remodeling in HGSOC, we established a CAF-associated mitochondrial metabolic signature, termed CMMS, based on 30 genes covering three functionally connected modules: contractile/myCAF activation, matrix remodeling/ECM organization, and mitochondrial metabolic regulation (Figure 1A). For each TCGA-HGSOC sample, ssGSEA scores were calculated for the three modules, z-transformed, and integrated into a composite CMMS score. A median-based threshold was applied to classify patients into CMMS-high and CMMS-low subgroups (Figure 1A). When TCGA-HGSOC samples were ranked according to CMMS, the 30-gene panel showed a clear expression gradient, particularly within the contractile and ECM-remodeling modules (Figure 1B). We further found that the 30 CMMS genes showed heterogeneous but evident tumor-associated expression alterations, particularly among contractile and ECM-remodeling genes (Figure S1A). These findings indicate that CMMS captures a coordinated stromal activation program characterized by myCAF-like contractility, matrix remodeling, and metabolic adaptation in HGSOC.

To clarify the biological composition of CMMS, we examined the contribution of each module to the composite score. CMMS was strongly correlated with the matrix-remodeling module and contractile/myCAF module, whereas its correlation with the mitochondrial/metabolic module was comparatively weaker (Figure 1C). Consistently, the contractile/myCAF and matrix-remodeling modules were highly correlated with each other, while the mitochondrial/metabolic module showed weak negative correlations with both modules (Figure 1D). These findings suggested that rather than representing a uniformly synchronized transcriptional program, CMMS captures a composite stromal state in which contractile activation and ECM remodeling form the dominant axis, while mitochondrial metabolic features provide a relatively auxiliary component and distinct dimension of CAF heterogeneity. The continuous distribution of CMMS scores across TCGA-HGSOC samples and two independent GEO cohorts (GSE32062 and GSE53963) supported its use as a quantitative stromal activation score for subsequent analyses (Figure 1E and Figure S1H,I).

The original CMMS score showed limited prognostic significance in the TCGA cohort. A LASSO–Cox model was further used to optimize the prognostic contribution of individual CMMS genes. Coefficient path and cross-validation analyses determined the optimal penalty parameter for constructing a LASSO-weighted CMMS score (Figure S1B–G). The LASSO-weighted CMMS effectively identified a poor-prognosis subgroup in TCGA-HGSOC, with higher risk scores corresponding to significantly poorer overall survival outcomes (HR = 1.3, 95% CI: 1.02–1.67, *p* = 0.036) (Figure 1F). Consistent prognostic trends were observed in the two GEO cohorts using cohort-specific LASSO-CMMS models, in which higher risk scores were associated with inferior survival outcomes (GSE32062: *p* < 0.001; GSE53963: *p* = 0.0198) (Figure 1G–I). To further address potential clinical confounding, we performed additional Cox regression analyses in TCGA-HGSOC using available clinicopathological variables. In univariable analysis, both the dichotomized LASSO-CMMS group and continuous LASSO-CMMS risk score were associated with overall survival (Figure S1J). In multivariable analysis adjusted for age, FIGO stage, and estimated tumor purity, the continuous LASSO-CMMS risk score remained significantly associated with poorer overall survival (HR = 1.196, 95% CI: 1.018–1.405, *p* = 0.0293; Tables S5 and S6). By contrast, estimated tumor purity was not significantly associated with overall survival in this model. Because residual disease, treatment history, platinum sensitivity, and BRCA/HRD status were unavailable or incompletely annotated in public cohorts, CMMS was interpreted as a prognostically relevant stromal signature rather than a definitive independent clinical prognostic factor.

To further characterize the biological relevance of CMMS, we evaluated its association with extracellular matrix (ECM) remodeling programs. Evaluated via an extended fibrosis-related gene signature, the ECM scores maintained a strong positive association with the CMMS. Although ECM-related genes are included in the CMMS construction, the ECM score was independently calculated using an expanded ECM gene set and showed a consistent association with CMMS. In TCGA-HGSOC, heatmap analysis demonstrated a clear gradient of ECM gene expression across samples ranked by CMMS, with key fibrotic markers including COL1A1, POSTN, LOX, and FN1 being progressively upregulated in the CMMS-high group (Figure 1I). This association was highly reproducible across TCGA (Spearman rho = 0.84, *p* < 0.001), GSE32062 (Spearman rho = 0.88, *p* < 0.001), and GSE53963 (Spearman rho = 0.72, *p* < 0.001), where CMMS strongly correlated with ECM scores (Figure 1J). This association was further supported by group-wise comparisons, showing consistently elevated ECM signature scores in CMMS-high tumors across the TCGA, GSE32062, and GSE53963 cohorts (Figure S1K–M). Consistently, CMMS was closely associated with representative matrix-remodeling genes, including COL1A1 (Spearman rho = 0.79, *p* < 0.001), POSTN (Spearman rho = 0.80, *p* < 0.001), and LOX (Spearman rho = 0.83, *p* < 0.001), in the TCGA cohort (Figure 1K). To address potential circularity in ECM-related analyses, we further performed overlap-controlled and leave-one-module-out sensitivity analyses. An overlap-free ECM score was generated using ECM-associated genes that were not included in the 30 CMMS genes. The original CMMS was strongly correlated with the overlap-free ECM score (Spearman rho = 0.894, *p* < 0.001) (Figure S2A,B). Importantly, this association remained significant after excluding the matrix-remodeling module from the CMMS calculation (CMMS_no_M: rho = 0.738, *p* < 0.001), although the correlation was attenuated. Similar associations were observed for CMMS_no_C and CMMS_no_Met (Figure S2C–E). These results indicate that the ECM-related signal of CMMS is not solely attributable to direct gene overlap but reflects a broader coordinated stromal remodeling program. In summary, our data suggest that the poor-prognosis CMMS-high subgroup is characterized by a robust fibrotic CAF-associated stromal remodeling phenotype.

### 2.2. CMMS Is Associated with Hypoxia-Dominant Metabolic Remodeling in HGSOC

**Figure 2:**
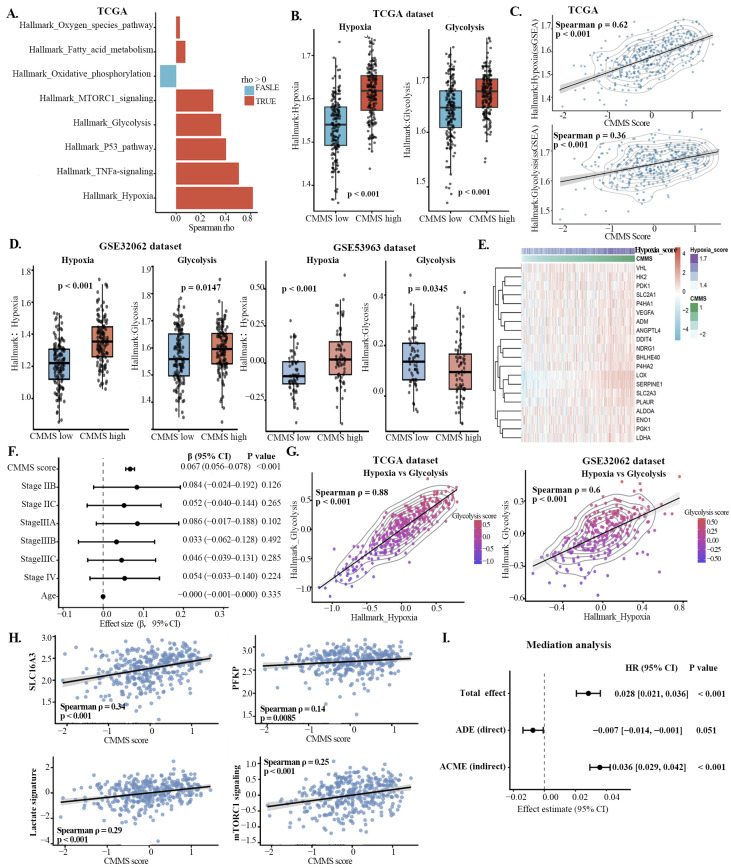
CMMS is associated with hypoxia-dominant metabolic remodeling in HGSOC. (**A**) Spearman correlation between CMMS score and selected hallmark metabolic or stress-related pathways in the TCGA-HGSOC cohort. (**B**) Comparison of hallmark hypoxia and glycolysis scores between CMMS-high and CMMS-low tumors in TCGA-HGSOC. (**C**) Correlations between CMMS score and hallmark hypoxia or glycolysis scores in TCGA-HGSOC. Spearman correlation coefficients and *p* values are shown. (**D**) Comparison of hallmark hypoxia and glycolysis scores between CMMS-high and CMMS-low tumors in GSE32062 and GSE53963 cohorts. (**E**) Heatmap showing the expression pattern of hypoxia-responsive genes in TCGA-HGSOC samples ranked by CMMS score. (**F**) Multivariable linear regression analysis evaluating the association between CMMS scores and hypoxia scores after adjustment for age and tumor stage. Effect sizes are shown as β coefficients with 95% confidence intervals. (**G**) Correlations between hallmark hypoxia and glycolysis scores in TCGA-HGSOC and GSE32062 cohorts. Spearman correlation coefficients and *p* values are shown. (**H**) Spearman correlations between CMMS scores and glycolysis- or lactate-related metabolic features, including SLC16A3, PFKP, lactate signature, and MTORC1 signaling, in TCGA-HGSOC. (**I**) Mediation analysis showing that tumor hypoxia largely mediates the correlation between CMMS and glycolysis.

We next investigated whether the fibrotic CMMS-high phenotype was accompanied by metabolic stress features commonly induced by dense stromal remodeling. In TCGA-HGSOC, hallmark pathway analysis identified hypoxia as the pathway most strongly correlated with CMMS, whereas oxidative phosphorylation showed an inverse association (Figure 2A). CMMS-high tumors consistently displayed elevated hypoxia scores (Spearman rho = 0.62, *p* < 0.001), with a more modest increase in glycolysis (Spearman rho = 0.36, *p* < 0.001) (Figure 2B,C). This hypoxia-dominant pattern was further validated in GSE32062 and GSE53963 (Figure 2D and Figure S1N,O).

At the gene level, CMMS-high tumors showed coordinated activation of hypoxia-responsive genes involved in angiogenesis, glycolysis, lactate handling, and matrix remodeling (Figure 2E). After adjustment in a multiple linear regression model, CMMS retained a significant positive association with the hypoxia program (β = 0.0672, *p* < 0.001) (Figure 2F). This indicates that higher CMMS scores are linked to greater hypoxia activation, independent of tumor stage. Other clinical factors such as age and tumor stage did not show significant effects on hypoxia (all *p* > 0.05) (Figure 2F). Hypoxia and glycolysis scores were strongly correlated in both the TCGA-HGSOC and GSE32062 cohorts (Spearman rho = 0.88 and 0.60, respectively; both *p* < 0.001; Figure 2G). Consistently, CMMS was positively associated with glycolysis- and lactate-related features, including SLC16A3, PFKP, the lactate signature, and mTORC1 signaling (Spearman rho = 0.14–0.34; all *p* < 0.01; Figure 2H). These findings suggest that CMMS-high tumors are accompanied by glycolytic/lactate-related metabolic features and mTORC1-associated activity. Mediation analysis demonstrated that the association between CMMS and glycolysis was largely mediated by tumor hypoxia (ACME = 0.036, *p* < 0.001), with negligible direct effects after adjustment, indicating a hypoxia-driven metabolic reprogramming downstream of fibrotic remodeling (Figure 2I). Together, these results suggest that CMMS-high tumors exhibit a hypoxia-centered stromal-metabolic phenotype in which glycolytic reprogramming is largely coupled to hypoxic stress.

### 2.3. CMMS Is Associated with CAF/TGFβ-Driven Immune Exclusion and Exhaustion-Prone T-Cell Immunity

**Figure 3:**
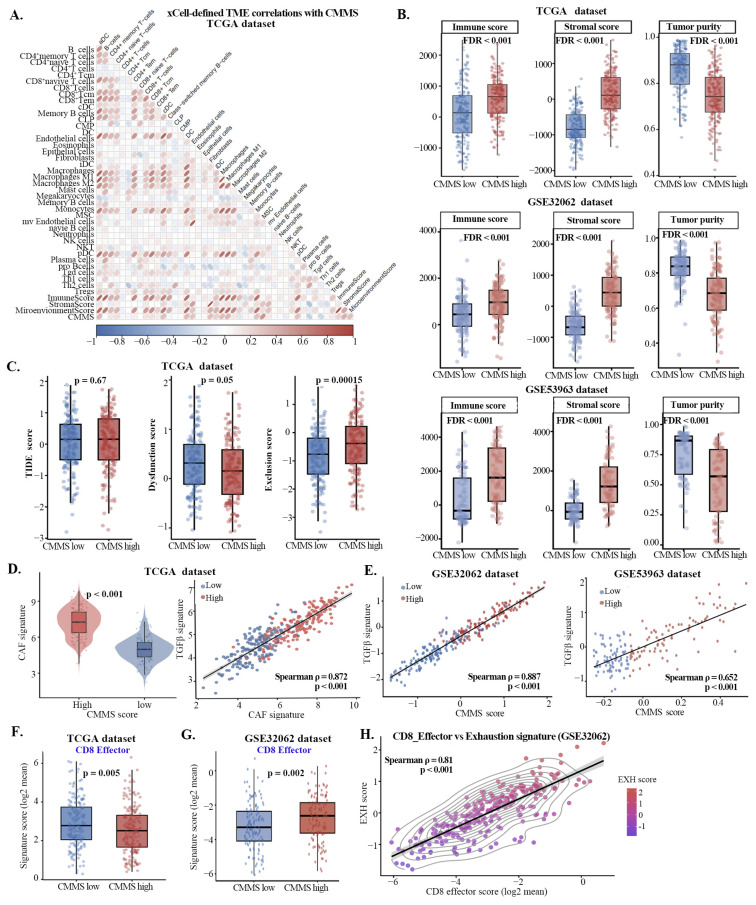
CMMS is associated with stromal-rich immune suppression in HGSOC. (**A**) xCell-based correlation matrix showing associations between CMMS score and tumor microenvironment cell types or microenvironment-related scores in the TCGA-HGSOC cohort. (**B**) Comparison of immune ESTIMATE between CMMS-high and CMMS-low tumors in TCGA-HGSOC, GSE32062, and GSE53963 cohorts. (**C**) Comparison of TIDE score, dysfunction score, and exclusion score between CMMS-high and CMMS-low tumors in the TCGA-HGSOC cohort. (**D**) Comparison of CAF signature scores between CMMS-high and CMMS-low tumors in TCGA-HGSOC, and correlation between CAF signature and TGFβ signature in the TCGA cohort. (**E**) Correlations between CMMS score and TGFβ signature in the GSE32062 and GSE53963 cohorts. (**F**) Comparison of CD8 effector signature scores between CMMS-high and CMMS-low tumors in TCGA-HGSOC. (**G**) Comparison of CD8 effector signature scores between CMMS-high and CMMS-low tumors in the GSE32062 cohort. (**H**) Association between CD8 effector score and exhaustion signature in GSE32062.

Having established that CMMS-high tumors display fibrotic and hypoxia-centered metabolic remodeling, we next examined whether this phenotype was associated with altered immune microenvironment features. We first evaluated the association between CMMS and immune infiltration using an independent deconvolution/enrichment method (xCell or CIBERSORT). xCell-based deconvolution indicated that CMMS-high tumors were enriched for stromal and immunoregulatory features, with positive associations observed for fibroblasts, M2-like macrophages, and regulatory T cells. Conversely, cytotoxic immune components, such as CD8^+^ T-cell and NK-cell signatures, decreased with increasing CMMS activity. In addition, CMMS was strongly associated with stromal-related scores, including the Stroma-Score and Microenvironment-Score, indicating that CMMS-high tumors are characterized by a stromal-enriched and immunosuppressive tumor microenvironment (Figure 3A). To improve the robustness of immune infiltration estimation, we further applied the ImmuCellAI algorithm for complementary analysis. CMMS showed positive correlations with immunoregulatory cell populations, including Tregs, Tr1 cells, and Th2 cells, whereas it was inversely related to cytotoxic immune populations such as CD8^+^ T, NK cells, and cytotoxic T lymphocytes (Figure S3A). ImmuCellAI-based deconvolution further supported the association between CMMS and immune microenvironment remodeling. Consistently, CMMS-high tumors showed significantly higher immune and stromal scores, together with reduced tumor purity, across the TCGA-HGSOC and two independent GEO cohorts (FDR < 0.001) (Figure 3B). These findings suggest that CMMS-high tumors are not immune-depleted but, instead, represent microenvironment-rich tumors with prominent stromal infiltration. Further analysis using TIDE-derived metrics showed that CMMS-high tumors had significantly elevated exclusion scores (*p* = 0.00015), whereas the overall TIDE score was not increased (*p* = 0.67), and dysfunction showed only a borderline difference (*p* = 0.05) (Figure 3C). Thus, CMMS appears to be preferentially associated with an immune-exclusion-associated phenotype rather than an immune-desert phenotype in HGSOC.

To further define the stromal program underlying the immune-exclusion-associated phenotype of CMMS-high tumors, we examined CAF and TGFβ-related signatures across the TCGA and GEO cohorts. In TCGA-HGSOC, CMMS-high tumors showed significantly higher CAF signature scores, and the CAF signature was strongly correlated with TGFβ signaling activity (Spearman rho = 0.872, *p* < 0.001) (Figure 3D). This association was consistently reproduced in the two independent GEO cohorts, where CMMS was positively correlated with the TGFβ signature in both the GSE32062 (Spearman rho = 0.887, *p* < 0.001) and GSE53963 cohorts (Spearman rho = 0.652, *p* < 0.001) (Figure 3E), supporting a conserved CAF/TGFβ stromal axis linked to an immune exclusion-related transcriptional pattern. Supplementary analyses further confirmed that CMMS was related to consistently elevated TGFβ activity across independent HGSOC cohorts (Figure S3B–D). CD8 effector activity was not uniformly depleted, with reduced effector signals in TCGA but increased effector signals in the GSE32062 and GSE53963 cohorts (Figure 3F,G and Figure S3E). In the latter cohort, CD8 effector activity was tightly coupled with the exhaustion signature (Spearman rho = 0.81, *p* < 0.001) (Figure 3H), suggesting that T-cell infiltration in CMMS-high tumors may occur in an exhaustion-prone state. These findings suggest that CMMS-high tumors may impair antitumor immunity through two non-mutually exclusive modes: CAF/TGFβ-associated immune exclusion and exhaustion-prone T-cell dysfunction in immune-infiltrated tumors.

### 2.4. CMMS Links Stromal Immune Exclusion-Associated Phenotype to Exhaustion-Prone T-Cell Dysfunction

**Figure 4:**
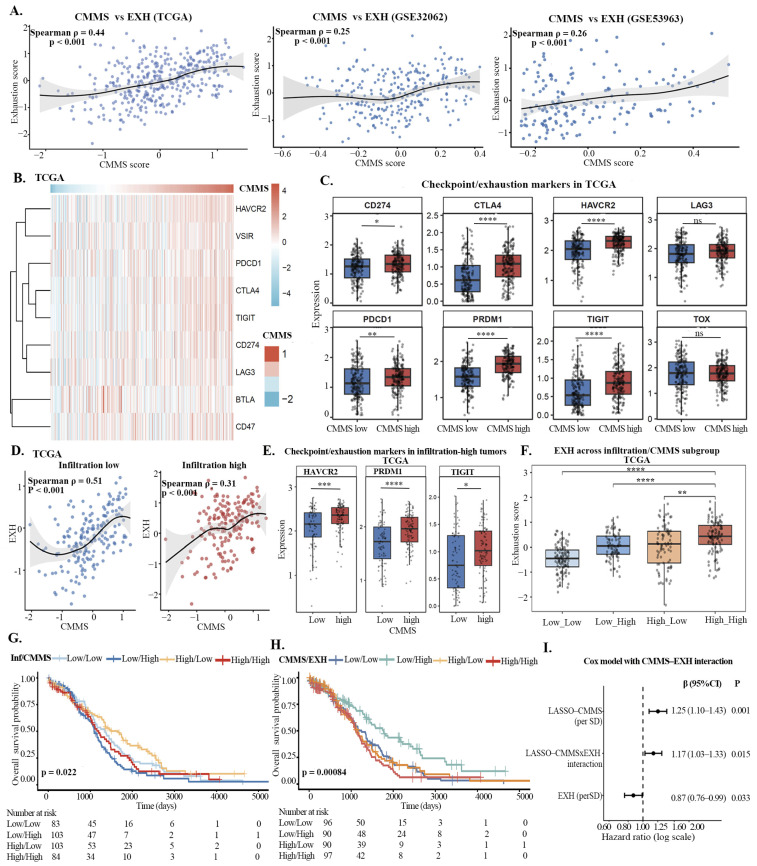
CMMS is associated with immune exclusion-associated phenotype, exhaustion-related dysfunction, and adverse prognosis in HGSOC. (**A**) Correlations between CMMS score and exhaustion signature in TCGA-HGSOC, GSE32062, and GSE53963 cohorts. Spearman correlation coefficients and *p* values are shown. (**B**) Heatmap showing the expression pattern of checkpoint and exhaustion-related genes in TCGA-HGSOC samples ranked by CMMS score. (**C**) Comparison of representative checkpoint/exhaustion marker expression between CMMS-high and CMMS-low tumors in the TCGA-HGSOC cohort. Group differences were evaluated using the Wilcoxon rank-sum test. * *p* < 0.05, ** *p* < 0.01, **** *p* < 0.0001; ns, not significant. (**D**) Correlations between CMMS score and exhaustion signature in infiltration-low and infiltration-high TCGA-HGSOC tumors. Spearman correlation coefficients and *p* values are shown. (**E**) Expression of exhaustion-associated checkpoint markers, including HAVCR2, PRDM1, and TIGIT, between CMMS-low and CMMS-high tumors within the infiltration-high subgroup. * *p* < 0.05, *** *p* < 0.001, **** *p* < 0.0001. (**F**) Exhaustion signature scores across combined infiltration/CMMS subgroups in TCGA-HGSOC. ns, not significant. ** *p* < 0.01, **** *p* < 0.0001. (**G**) Kaplan–Meier survival assessment for TCGA-derived HGSOC patients categorized by immune infiltration status and LASSO-weighted CMMS risk score. (**H**) Kaplan–Meier survival analysis of TCGA-HGSOC patients stratified by LASSO-weighted CMMS risk score and exhaustion signature. (**I**) Cox proportional hazards model evaluating the main effects of CMMS and exhaustion signature, together with their interaction term. Hazard ratios are shown per standard deviation increase.

Given the coexistence of CD8 effector and exhaustion signatures in CMMS-high tumors, we further explored whether the infiltrated immune component of CMMS-high tumors was accompanied by exhaustion and checkpoint activation. Across the TCGA-HGSOC and two independent GEO cohorts, the CMMS score was positively correlated with the exhaustion signature, with the strongest association observed in TCGA (Spearman rho = 0.44, *p* < 0.001) and consistent positive trends in GSE32062 (Spearman rho = 0.25, *p* < 0.001) and GSE53963 (Spearman rho = 0.26, *p* < 0.001) (Figure 4A). In TCGA-HGSOC, heatmap analysis showed a coordinated increase in immune checkpoint and exhaustion-related genes along the CMMS gradient (Figure 4B). Consistently, CMMS-high tumors displayed higher expression of a few inhibitory immune markers, including CD274, CTLA4, HAVCR2, PDCD1, PRDM1, and TIGIT, whereas LAG3 and TOX showed no significant difference (Figure 4C). This pattern suggests that CMMS-high tumors are associated with retained but functionally constrained effector immune activity and an exhaustion-prone T-cell state.

Since exhaustion signatures may partly reflect the abundance of infiltrating T cells, we next stratified tumors by immune infiltration status to determine whether CMMS was associated with exhaustion within infiltrated tumors. CMMS remained positively correlated with the exhaustion score in both infiltration-low and infiltration-high tumors, indicating that the CMMS–exhaustion relationship was not merely driven by differences in immune abundance (Figure 4D). Notably, within infiltration-high tumors, CMMS-high samples showed increased expression of exhaustion-associated checkpoint markers, including HAVCR2, PRDM1, and TIGIT (*p* < 0.05) (Figure 4E). Combined stratification by infiltration and CMMS further revealed that tumors with both high infiltration and high CMMS exhibited the highest exhaustion scores (*p* < 0.001) (Figure 4F). These findings suggest that, beyond its association with an immune exclusion-associated phenotype, CMMS may also mark an exhaustion-prone immune state in tumors with retained immune infiltration.

We then examined whether the interplay between CMMS, immune infiltration, and exhaustion was reflected in patient outcome. Joint survival analyses further linked the CMMS-associated immune phenotype to clinical outcome. This analysis suggested that immune infiltration was clinically beneficial only when the stromal CMMS program was low; in CMMS-high tumors, infiltrating immune signals appeared to be insufficient to translate into improved survival (*p* = 0.022) (Figure 4G). Similarly, CMMS/EXH stratification revealed that the exhaustion signature had distinct prognostic implications depending on the CMMS status: EXH-high tumors showed relatively favorable survival when CMMS was low but poor survival when CMMS was high (*p* = 0.00084) (Figure 4H). A Cox model confirmed a significant CMMS–EXH interaction, indicating that CMMS modifies the prognostic impact of exhaustion-related immune signals (*p* = 0.015) (Figure 4I). Together, these findings suggest that CMMS-high stromal remodeling is associated with an immune exclusion-associated phenotype and an altered prognostic context of infiltrating or exhaustion-prone immune states, reflecting a dysfunctional and clinically unfavorable tumor immune phenotype.

### 2.5. CMMS Is Predominantly Enriched in CAFs and Marks a myCAF-like ECM-Remodeling State

**Figure 5:**
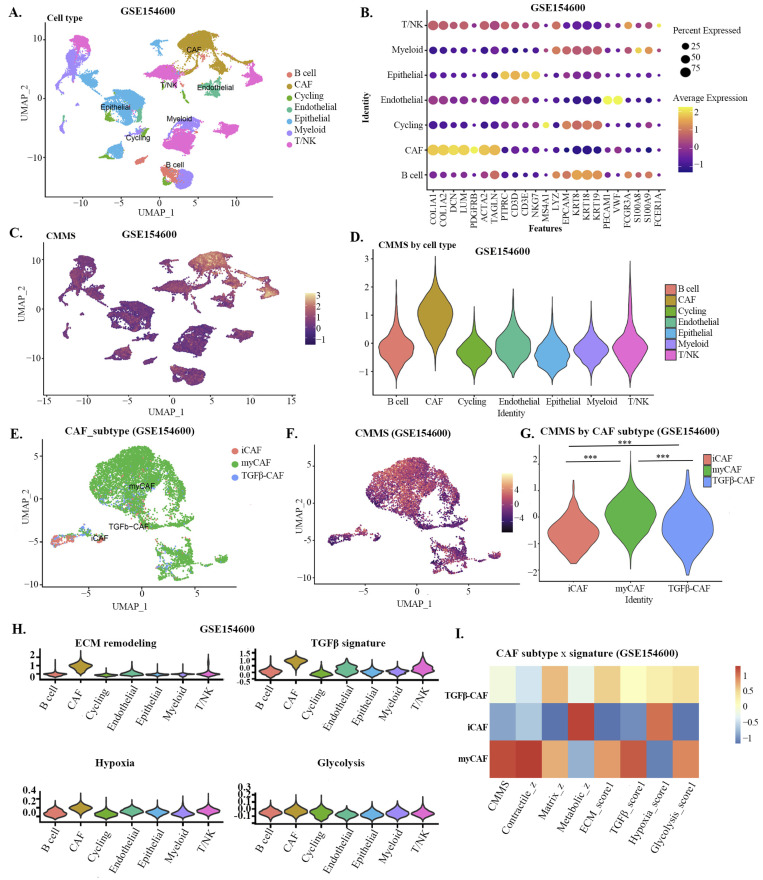
Single-cell analysis identifies CAFs as the major cellular source of CMMS activity in HGSOC. (**A**) UMAP visualization of major cell populations in the GSE154600 single-cell RNA-seq dataset. (**B**) Dotplot of canonical lineage markers across annotated cell types. (**C**) FeaturePlot showing the distribution of CMMS scores across the single-cell UMAP. (**D**) Violin plot comparing CMMS scores among major cell types. (**E**) UMAP visualization of CAF subtypes in GSE154600, including iCAF, myCAF, and TGFβ-CAF subsets. (**F**) FeaturePlot showing CMMS score distribution within the CAF compartment. (**G**) Violin plot comparing CMMS scores among CAF subtypes. *** *p* < 0.001. (**H**) Violin plots showing ECM-remodeling, TGFβ, hypoxia, and glycolysis signature scores across major cell types in GSE154600. (**I**) Heatmap summarizing CMMS and related functional signatures across CAF subtypes.

To clarify whether the CMMS-associated stromal and immune phenotypes were driven by specific cell populations, we analyzed CMMS activity at the single-cell resolution. We next used the GSE154600 scRNA-seq data to trace the cellular origin of CMMS activity. Quality-control assessment of the GSE154600 single-cell dataset showed consistent RNA feature/count distributions across samples, low mitochondrial and hemoglobin transcript proportions, and high post-filtering cell retention rates, supporting the reliability of downstream single-cell analyses (Figure S3F–H). After applying quality control filtering, a total of 47,706 high-quality cells were retained across five HGSOC samples. Major cell populations, including CAFs, epithelial cells, T/NK cells, myeloid cells, endothelial cells, B cells, and cycling cells, were identified and visualized by UMAP (Figure 5A). Stacked bar plots revealed substantial heterogeneity in the abundance of CAFs, immune cells, and epithelial cells, highlighting marked differences in the tumor microenvironment architecture across patients (Figure S4A). Cell populations were assigned based on established lineage markers, with CAFs marked by fibroblast and collagen genes, epithelial cells by EPCAM/KRT expression, T/NK cells by CD3D/CD3E/NKG7, myeloid cells by LYZ/MS4A7, endothelial cells by PECAM1/VWF, and B cells by MS4A1 (Figure 5B). Projection of the CMMS activity onto the UMAP revealed a preferential enrichment of the CMMS score within the CAF compartment (Figure 5C). Consistently, violin plot analysis confirmed that CAFs displayed the highest CMMS activity among all major cell types (Figure 5D). These results indicate that CMMS is predominantly a CAF-enriched stromal program at the single-cell level.

We further dissected CMMS activity within the CAF compartment. CAFs in GSE154600 were annotated into iCAF, myCAF, and TGFβ-CAF subsets (Figure 5E). Visualization of CMMS on the CAF UMAP embedding revealed a distinct spatial pattern. CMMS-high cells were predominantly localized within myCAF clusters and extended into regions corresponding to TGFβ-activated CAFs, forming a continuous gradient across CAF states. In contrast, iCAFs consistently exhibited low CMMS levels (Figure 5F). Pairwise Wilcoxon rank-sum tests identified substantial differences in CMMS scores among CAF subtypes. myCAFs harbored significantly higher CMMS scores than both iCAFs and TGFβ-CAFs, with adjusted *p* values below 2 × 10^−16^ for both comparisons. Moreover, TGFβ-CAFs displayed markedly elevated CMMS scores relative to iCAFs (adjusted *p* = 1.1 × 10^−6^) (Figure 5G). These findings suggest that CMMS reflects a continuous transcriptional program associated with contractile and matrix-producing CAF states rather than a discrete CAF subtype. Consistent with this pattern, ECM-remodeling and TGFβ-related signatures were predominantly enriched in CAFs among major cell types, while hypoxia and glycolysis showed weaker and more heterogeneous distributions at the single-cell level (Figure 5H). CAF subtype-level signature analysis further showed that myCAFs were enriched for CMMS, contractile activation, ECM-remodeling, and TGFβ-related programs (Figure 5I). These findings indicate that CMMS mainly reflects a myCAF-like ECM-remodeling CAF state rather than a uniform signal across all CAF populations. In support of this, CMMS-high CAFs exhibited elevated expression of contractile and ECM-remodeling markers such as COL11A1, CNN1, ACTG2, MFAP5, TAGLN, and POSTN, further confirming their activated myCAF-like phenotype (Figure S2B,C).

### 2.6. CMMS-High CAFs Communicate with T Cells Through ECM- and Chemokine-Related Signaling

**Figure 6:**
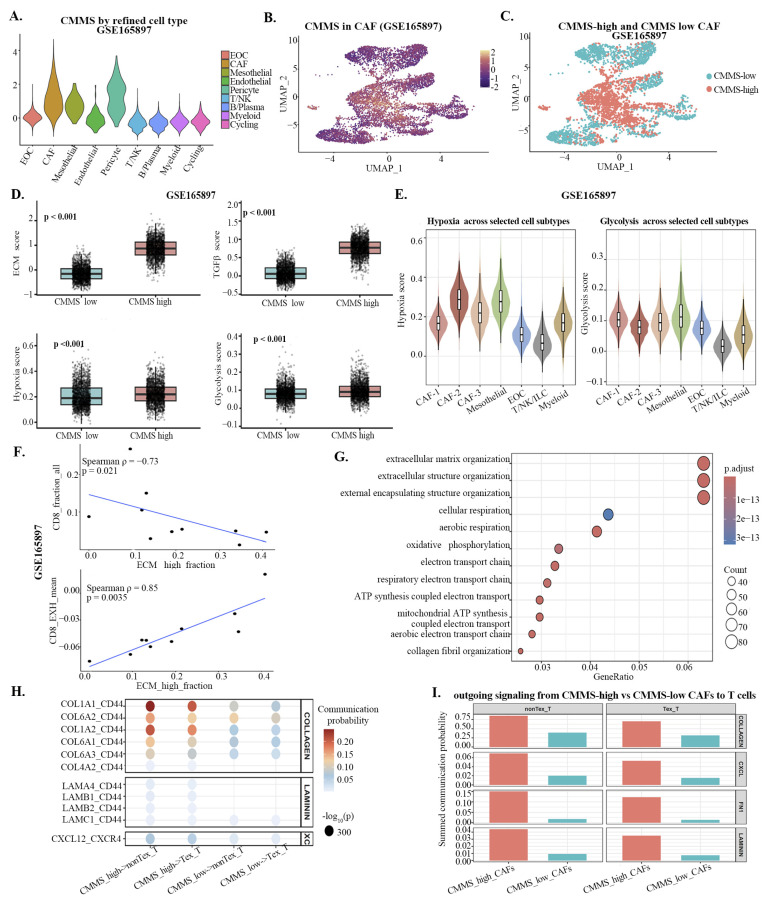
Independent single-cell validation of CMMS-high CAFs and their association with CD8 exclusion and exhaustion. (**A**) Violin plot showing CMMS scores across refined cell types in the GSE165897 scRNA-seq cohort. (**B**) FeaturePlot showing CMMS score distribution within the CAF compartment. (**C**) UMAP visualization of CMMS-high and CMMS-low CAFs. (**D**) Comparison of ECM-remodeling, TGFβ, hypoxia, and glycolysis scores between CMMS-high and CMMS-low CAFs. (**E**) Violin plots showed hypoxia and glycolysis signature scores across selected CAF, mesothelial, epithelial, T/NK/ILC, and myeloid cell subsets. (**F**) Sample-level correlations between ECM-high CAF fraction and CD8 T-cell fraction or CD8 exhaustion score. (**G**) GO enrichment analysis of genes upregulated in CMMS-high CAFs compared with CMMS-low CAFs in GSE165897. (**H**) Ligand–receptor interaction analysis showing matrix- and chemokine-related communication from CMMS-high or CMMS-low CAFs to exhausted and non-exhausted T-cell subsets. (**I**) Summed outgoing signaling probability from CMMS-high and CMMS-low CAFs toward exhausted and non-exhausted T-cell subsets across selected signaling pathways.

We further validated the single-cell findings in an independent HGSOC scRNA-seq cohort, GSE165897. The GSE165897 validation scRNA-seq dataset passed standard quality-control filtering and was annotated into major tumor and microenvironmental cell populations (Figure S4D,E). Consistent with GSE154600, CMMS activity was preferentially enriched in stromal populations, particularly CAFs and pericyte-like cells, whereas epithelial and immune compartments showed lower CMMS scores (Figure 6A). Within the CAF compartment, CMMS activity displayed a heterogeneous spatial distribution, allowing the identification of CMMS-high and CMMS-low CAF states (Figure 6B,C). Functionally, CMMS-high CAFs exhibited markedly elevated ECM-remodeling and TGFβ signatures, together with increased hypoxia and glycolysis scores (*p* < 0.001) (Figure 6D). CMMS activity in GSE165897 was enriched in stromal compartments and strongly correlated with ECM and TGFβ signatures, independently validating CMMS as a CAF-associated matrix-remodeling program (Figure S4G,H). The hypoxia and glycolysis scores were preferentially enriched in stromal-related compartments, particularly CAF and mesothelial populations, while EOC cells showed intermediate activity and T/NK/ILC cells remained low. This pattern suggests that the CMMS-associated hypoxic–glycolytic phenotype is not purely tumor cell-intrinsic but is closely coupled to stromal remodeling and myofibroblastic CAF activation (Figure 6E and Figure S4I). At the sample level, a higher fraction of ECM-high CAFs was associated with a reduced CD8 T-cell fraction (Spearman rho = −0.73, *p* = 0.021) but increased CD8 exhaustion activity (Spearman rho = 0.85, *p* = 0.0035) (Figure 6F). To reduce pseudo-replication in the single-cell analysis, we further aggregated CAF and CD8 T-cell metrics at the patient level in GSE165897. Across patients with sufficient CAF and CD8 T-cell numbers, the mean CAF CMMS activity showed a positive but non-significant trend with the mean CD8 exhaustion score (Spearman rho = 0.418, *p* = 0.229; *n* = 10 patients) (Figure S4F). These results further support that CMMS-high CAFs represent an ECM/TGFβ-activated stromal population associated with immune exclusion-related features and exhaustion-prone T-cell dysfunction.

To further characterize the functional programs of CMMS-high CAFs in the validation scRNA-seq cohort, we performed enrichment and cell–cell communication analyses. CMMS-high CAFs displayed a transcriptional program centered on matrix remodeling, with enriched GO terms related to ECM organization, extracellular structure assembly, and collagen fibril formation, as well as mitochondrial respiratory processes, including oxidative phosphorylation and electron transport chain activity (Figure 6G). Supporting these findings, CMMS-high CAFs showed elevated expression of contractile and matrix-remodeling genes and were enriched for EMT/myogenesis-related hallmark programs, suggesting that CMMS-high CAFs represent a matrix-remodeling, myofibroblastic, and stress-adapted stromal state (Figure S5A,B). Ligand–receptor analysis further revealed enhanced matrix- and chemokine-related interactions from CMMS-high CAFs toward both exhausted and non-exhausted T-cell subsets, particularly involving collagen-CD44, laminin-related, and CXCL12-CXCR4 signaling axes (Figure 6H). Summed outgoing signaling analysis confirmed that CMMS-high CAFs exhibited stronger COLLAGEN, FN1, LAMININ, and CXCL signaling toward T cells than CMMS-low CAFs (Figure 6I). Ligand–receptor-related expression patterns further confirmed the availability of matrix and chemokine signaling components in CMMS-high CAFs and T-cell subsets (Figure S5C). These findings suggest that CMMS-high CAFs may shape the T-cell microenvironment through ECM-rich and chemokine-mediated communication programs.

### 2.7. Translational and Experimental Validation of the CMMS-Associated Immune-Suppressive Stromal Phenotype

**Figure 7:**
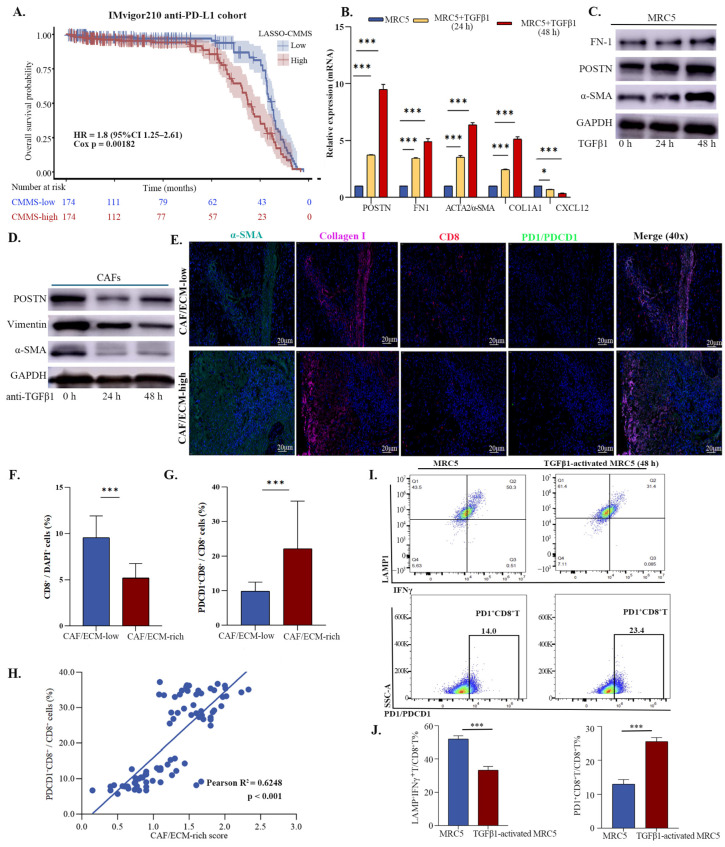
CMMS-like stromal activation drives matrix remodeling and exhaustion-prone CD8^+^ T-cell dysfunction. (**A**) Kaplan–Meier analysis of overall survival in the IMvigor210 cohort stratified by LASSO-weighted CMMS risk score. Hazard ratio was estimated using Cox proportional hazards regression. (**B**) qRT-PCR analysis of representative CMMS-related genes in MRC5 fibroblasts treated with TGFβ1 for 24 or 48 h. Differences were assessed via one-way ANOVA or two-tailed *t*-test. * *p* < 0.05, *** *p* < 0.001. (**C**) Western blot analysis of representative CMMS-related genes in MRC5 fibroblasts treated with TGFβ1 for 48 h. (**D**) Western blot analysis of Vimentin, POSTN, and α-SMA protein expression in CAFs treated with anti-TGFβ1 neutralizing antibody for 0, 24, and 48 h. (**E**) Representative multiplex immunofluorescence images of CMMS-low and CMMS-high HGSOC tissues stained for α-SMA, Collagen I, CD8, and PDCD1. Scale bar, 20 μm. (**F**) CD8^+^ T-cell proportion across CMMS-like CAF/ECM-rich regions and CAF/ECM-low regions. *** *p* < 0.001. (**G**) PDCD1+CD8^+^ T-cell proportion across CMMS-like CAF/ECM-rich regions and CAF/ECM-low regions. *** *p* < 0.001. (**H**) Correlation between CAF/ECM-rich score and the proportion of PDCD1^+^CD8^+^ T cells in HGSOC tissues. CAF/ECM-rich score was calculated as the mean of the z-normalized α-SMA^+^ area and Collagen I^+^ area. Each dot represents one case. (**I**) Representative flow cytometry plots showing LAMP1^+^IFNγ^+^ and PD1^+^ populations among CD8^+^ T cells after co-culture with control MRC5 cells or TGFβ1-activated MRC5 cells. (**J**) Quantification of LAMP1^+^IFNγ^+^CD8^+^ T cells and PD1^+^CD8^+^ T cells. Data are presented as mean ± SEM. Statistical significance was determined using a two-sided Student’s *t* test. *** *p* < 0.001.

Finally, we performed an exploratory analysis in the anti-PD-L1-treated IMvigor210 cohort to assess whether CMMS-related stromal features might be associated with immunotherapy outcomes in a non-HGSOC setting. Patients with higher LASSO-weighted CMMS scores showed shorter overall survival than those with lower scores (HR: 1.8, 95% CI: 1.25–2.61, *p* = 0.00182) (Figure 7A). Given that IMvigor210 is not an ovarian cancer cohort, this result was interpreted as hypothesis-generating evidence suggesting a possible link between CMMS-like stromal remodeling and unfavorable outcome after immune checkpoint blockade, rather than direct validation of immunotherapy resistance in HGSOC.

To experimentally validate whether TGFβ signaling could induce a CMMS-like fibroblast activation state, MRC5 fibroblasts were stimulated with TGFβ1 for 24 or 48 h as a standardized fibroblast activation model. TGFβ1 stimulation of MRC5 fibroblasts induced a CMMS-like activated stromal phenotype, with robust upregulation of POSTN, FN1, ACTA2/α-SMA, and COL1A1, particularly after 48 h (Figure 7B). CXCL12 showed an opposite trend, indicating that the chemokine component may be uncoupled from the core TGFβ-induced contractile–ECM-remodeling program. Consistently, Western blot analysis revealed elevated protein expression levels of POSTN, α-SMA and FN1 in MRC5 cells following 48 h of TGFβ stimulation (Figure 7C). Conversely, anti-TGFβ1 treatment attenuated the activated CAF phenotype, as indicated by reduced Vimentin, POSTN, and α-SMA protein levels over time (Figure 7D). These data suggest that TGFβ signaling contributes to the maintenance of a CMMS-like ECM-remodeling CAF state.

Multiplex immunofluorescence showed that CMMS-high tissues exhibited enhanced α-SMA^+^ CAF activation and Collagen I deposition compared with CMMS-low tissues. CD8^+^ T cells appeared spatially associated with CAF/ECM-rich regions and showed stronger PDCD1-related signals, supporting a tissue-level link between CMMS-like stromal remodeling and exhaustion-prone CD8^+^ T-cell states (Figure 7E). CAF/ECM-high regions showed reduced CD8^+^ T-cell accumulation and a higher fraction of PDCD1^+^CD8^+^ cells among residual CD8^+^ T cells, supporting a CAF/ECM-associated immune-restrictive and exhaustion-prone microenvironment (*p* < 0.001) (Figure 7F,G). Tissue-level multiplex immunofluorescence further supported a spatial association between CAF/ECM-rich regions and the CD8^+^ T-cell distribution or PDCD1^+^CD8^+^ T-cell enrichment, providing complementary tissue-level evidence for a CAF/ECM-associated immune context. To assess whether CMMS-like stromal remodeling was associated with exhaustion-prone CD8^+^ T-cell states in situ, we quantified a CAF/ECM-rich score based on α-SMA and Collagen I positivity and correlated it with the proportion of PDCD1^+^CD8^+^ T cells (Pearson R^2^ = 0.6248, *p* < 0.001) (Figure 7H). The CAF/ECM-rich score was positively correlated with the PDCD1^+^CD8^+^ proportion, indicating that stromal regions with stronger CAF activation and ECM deposition were associated with increased exhaustion marker expression in CD8^+^ T cells. Functionally, co-culture with TGFβ1-activated MRC5 (a-MRC5) fibroblasts reduced LAMP1^+^IFNγ^+^CD8^+^ T cells and increased PD1^+^CD8^+^ T cells, indicating impaired effector activity and an exhaustion-prone phenotype (*p* < 0.001) (Figure 7I,J). These data support a functional link between CMMS-like fibroblast activation and CD8^+^ T-cell dysfunction.

## 3. Discussion

This study identifies CMMS as a CAF-enriched stromal remodeling program associated with poor prognosis and impaired antitumor immunity in HGSOC. Unlike conventional stromal scores that mainly reflect fibroblast abundance or ECM gene expression, CMMS integrates contractile CAF activation, matrix remodeling, and mitochondrial metabolic adaptation. Our analyses across bulk and single-cell cohorts suggest that CMMS-high tumors represent a fibrotic and hypoxia-associated stromal phenotype accompanied by an unfavorable immune context, rather than a simple high-stroma phenotype. This concept is consistent with previous studies showing that TGFβ-activated fibroblastic stroma and cancer-associated ECM programs can favor immune escape and substantially curtail clinical responses to anti-PD-1/PD-L1 treatment [21,22,23].

CAFs comprise diverse functional subtypes, including myCAF, iCAF and antigen-presenting CAF states [24,25]. The dominant biological axis of CMMS was contractile–ECM remodeling. The contractile/myCAF and matrix-remodeling modules were tightly coupled, whereas the mitochondrial/metabolic module showed a relatively distinct and weaker association with the composite CMMS phenotype. These findings indicate that CMMS should be interpreted as a composite CAF-associated stromal state, in which myofibroblast activation and ECM remodeling form the structural backbone, while mitochondrial/metabolic features capture additional CAF-state heterogeneity rather than constituting an independent dominant axis. This ECM-dominant interpretation was supported by the strong association of CMMS with ECM scores and matrix-remodeling genes across independent cohorts, as well as by overlap-controlled and leave-one-module-out analyses. Biologically, this interpretation is consistent with prior evidence that collagen density and matrix organization can shape tumor-infiltrating T-cell activity and spatial accessibility [7].

A key finding is the link between CMMS and hypoxia-centered metabolic remodeling. Hypoxia was the hallmark pathway most strongly associated with CMMS, and mediation analysis suggested that hypoxia largely accounted for the relationship between CMMS and glycolysis. Single-cell analysis further showed that the hypoxia and glycolysis programs were enriched in stromal-related compartments, supporting a multicellular stromal contribution to the metabolic phenotype observed in bulk tumors. Thus, glycolytic reprogramming in CMMS-high tumors may arise from stromal remodeling-induced hypoxic stress rather than from a purely tumor cell-intrinsic metabolic shift. This interpretation is biologically plausible, as hypoxia and metabolic stress have been shown to reinforce exhaustion-related T-cell states, while lactate-rich and metabolically competitive tumor microenvironments can blunt T- and NK-cell function [13,15,26].

CAFs and the stromal matrix have been implicated in T-cell exclusion and resistance to immunotherapy [27,28]. CMMS was also closely connected to immune remodeling. CMMS-high tumors showed increased immune and stromal scores but reduced tumor purity, indicating that they were not simply immune-desert tumors. Instead, CMMS was preferentially associated with TIDE-derived immune exclusion-associated phenotype and a conserved CAF/TGFβ axis. This aligns with the TIDE framework, which models immune escape through two major mechanisms: T-cell dysfunction in infiltrated tumors and T-cell exclusion in tumors with impaired T-cell infiltration [29]. TGFβ signaling in stromal cells has been shown to restrict T-cell penetration and attenuate the response to PD-L1 blockade [21]. These findings suggest that CMMS-high tumors may establish a stromal barrier that limits productive T-cell access. In parallel, CMMS was linked to exhaustion-related immune dysfunction. CD8 effector signals were not uniformly depleted; however, when present, they were strongly coupled with exhaustion signatures. This supports a dual model in which CMMS-high tumors suppress antitumor immunity through both the stromal immune exclusion-associated phenotype and exhaustion-prone dysfunction in infiltrated tumors. This interpretation is consistent with the current view that exhaustion is a coordinated transcriptional and functional state rather than the expression of any single inhibitory receptor. It is also compatible with HGSOC-specific evidence showing that exhausted CD8^+^ T-cell states may retain cytotoxic features in certain spatial contexts [30].

Several CAF- or stroma-related prognostic signatures have been reported in ovarian cancer. For example, a POSTN/TGFBI-associated stromal signature was previously shown to predict poor prognosis in serous epithelial ovarian cancer, supporting the clinical relevance of ECM-rich stromal programs. Subsequent studies further linked distinct CAF functional states, fibroblast modules, and CAF-associated paracrine signaling to ovarian cancer prognosis, immune microenvironment remodeling, and therapy response [31,32]. More recently, integrative single-cell and bulk transcriptomic analyses have generated CAF-related indices for predicting prognosis and immune contexture in ovarian cancer. Compared with these studies, CMMS provides a biologically interpretable framework that integrates contractile/myCAF activation, ECM-remodeling, and mitochondrial/metabolic CAF-state features. Importantly, our module-level analyses indicate that the dominant biological axis of CMMS is contractile–ECM remodeling, whereas the mitochondrial/metabolic component captures additional CAF-state heterogeneity rather than an independent dominant axis. Thus, CMMS should be considered an ECM/myCAF-dominant stromal remodeling signature that complements, rather than replaces, previously reported CAF or fibrosis-related prognostic signatures.

The clinical relevance of this immune remodeling was supported by survival analyses. The original CMMS score showed limited prognostic value, suggesting that equal weighting of its components may dilute its biological impact. In contrast, the LASSO-derived model assigns differential weights, improving prognostic performance. Moreover, the prognostic impact of immune infiltration and exhaustion appeared to depend on CMMS status. Higher levels of immune infiltration predicted better survival outcomes mainly under low CMMS conditions, whereas CMMS-high tumors showed poor outcomes despite immune infiltration. The significant CMMS–EXH interaction further suggests that CMMS modifies the clinical meaning of exhaustion-related immune signals, potentially converting immune activation into a dysfunctional and unfavorable state.

Single-cell validation localized CMMS activity predominantly to CAFs, especially myCAF-like subsets with contractile, ECM-remodeling, and TGFβ-related programs. In the independent GSE165897 cohort, CMMS-high CAFs retained ECM/TGFβ activation and were associated with reduced CD8 abundance and increased CD8 exhaustion at the sample level. Cell–cell communication analysis further suggested enhanced matrix- and chemokine-related signaling from CMMS-high CAFs toward T-cell subsets, including COLLAGEN, FN1, LAMININ, and CXCL pathways. The CXCL-related component is particularly relevant given previous evidence that CAF-derived CXCL12 can mediate T-cell exclusion and limit anti-PD-L1 efficacy [33]. These results provide cellular support for the stromal–immune model inferred from bulk transcriptomes.

Importantly, our experimental validation further supported the spatial and functional relevance of the CMMS model. Multiplex immunofluorescence showed that α-SMA^+^/COL1A1^+^ stromal regions were associated with spatial restriction of CD8^+^ T cells and increased exhaustion marker expression among CD8^+^ cells. TGFβ has been shown to induce CAF-associated programs in advanced high-grade serous ovarian tumors [34]. In vitro, TGFβ-activated fibroblasts acquired a CMMS-like contractile and matrix-remodeling phenotype and were associated with exhaustion-related marker expression in CD8^+^ T cells. These experimental findings strengthen the link between CMMS-like CAF activation, stromal immune exclusion, and exhaustion-prone T-cell dysfunction.

The exploratory IMvigor210 analysis suggests that CMMS-associated stromal remodeling may also be relevant to immunotherapy resistance, as high LASSO-weighted CMMS was associated with an inferior outcome after anti-PD-L1 therapy. This observation is consistent with the original IMvigor210-based analysis showing that TGFβ activity in fibroblastic stroma was associated with T-cell exclusion and lack of response to anti-PD-L1 therapy [21]. Although this finding requires cautious interpretation because IMvigor210 is not an ovarian cancer cohort, it is consistent with the concept that stromal exclusion and TGFβ-rich microenvironments can limit checkpoint blockade efficacy. Therapeutically, CMMS-high tumors may require stromal remodeling strategies, such as targeting TGFβ signaling, ECM organization, LOX-mediated matrix stiffening, CXCL12-CXCR4 signaling, or hypoxia/lactate-associated metabolic stress, in combination with chemotherapy or immune checkpoint blockade. However, targeting CAF-associated stromal remodeling remains challenging. CAFs are highly heterogeneous, and some fibroblast populations may exert tumor-restraining functions depending on the context. Broad depletion of CAFs or nonspecific suppression of stromal programs may therefore produce unintended effects. In addition, ECM remodeling is spatially heterogeneous and may require biomarker-guided patient stratification. Thus, CMMS may help identify tumors with ECM/myCAF-dominant stromal remodeling, but its therapeutic utility will require prospective validation and functional testing in ovarian cancer-specific models.

A limitation of our immune-exclusion analysis is that bulk RNA-seq deconvolution and TIDE-based exclusion scores cannot directly demonstrate spatial exclusion of immune cells from tumor nests. Although our integrative analyses link CMMS to stromal remodeling, immune exclusion-related programs, and exhaustion-prone CD8^+^ T-cell states, these findings should not be interpreted as definitive evidence that CMMS-high CAFs directly cause immune exclusion-associated phenotype or checkpoint resistance. Bulk transcriptomic deconvolution, module scoring, single-cell correlation analyses, and mediation-type models are inherently associative. Our in vitro assays provide functional support that activated fibroblasts can impair CD8^+^ T-cell effector activity, but they do not fully recapitulate the spatial and cellular complexity of HGSOC tissues. Future studies using spatially resolved perturbation models, CAF-specific genetic manipulation, and in vivo immune-competent systems will be required to determine whether CMMS-associated CAF programs directly regulate CD8^+^ T-cell exclusion, dysfunction, or therapeutic resistance.

Because CMMS reflects stromal and ECM-enriched transcriptional programs, its associations with prognosis and immune features may be influenced by tumor purity, stromal content, and differences in tissue composition. Although we performed tumor-purity-adjusted analyses in TCGA-HGSOC where possible, residual confounding cannot be excluded.

Although single-cell data identified CMMS-high CAF states, direct spatial and functional validations remain necessary. Recent spatial studies in HGSOC further emphasize that the stromal and immune compartments are organized into distinct spatial niches, reinforcing the need for tissue-level validation of the CMMS-associated immune exclusion-associated phenotype [35]. In addition, the IMvigor210 analysis should be viewed as exploratory because it was performed in a non-ovarian anti-PD-L1-treated cohort. Future multiplex immunofluorescence, spatial profiling, and CAF–T-cell functional assays will be important to validate whether CMMS-like CAFs directly contribute to the immune exclusion-associated phenotype and T-cell exhaustion.

Despite the wide usage of TGFβ1-stimulated MRC-5 cells for modeling fibroblast activation, this cell line fails to recapitulate the heterogeneous landscape of primary HGSOC CAFs. Although primary ovarian CAFs confer higher clinical authenticity to our experiments, patient-matched CAF perturbation analyses are still required to uncover the functional uniqueness of CMMS-high CAF phenotypes. In addition, targeted perturbation of the ECM/TGFβ/chemokine pathways and immune-competent in vivo systems will be required to establish causal mechanisms.

In summary, CMMS defines a CAF-associated stromal state linking ECM remodeling, hypoxic metabolic stress, the immune exclusion-associated phenotype, and exhaustion-prone T-cell dysfunction in HGSOC. These findings provide a conceptual framework for understanding stromal barriers to antitumor immunity and suggest that CMMS may help guide stromal-targeted immunotherapeutic strategies.

## 4. Materials and Methods

### 4.1. Data Collection and Preprocessing

Gene expression profiles and matched clinical annotations for ovarian cancer were downloaded from publicly accessible resources, including TCGA-OV and the GEO datasets GSE32062 and GSE53963. Only samples with available overall survival information were retained for prognostic analyses. For TCGA-OV, cases annotated as serous ovarian carcinoma were used to represent the HGSOC cohort. Gene expression profiles were processed at the gene-symbol level. For RNA-seq data, expression values were log-transformed after normalization. In microarray datasets, platform-specific annotation files were used to match probe identifiers to gene symbols. When several probes corresponded to the same gene, the probe showing the greatest average expression was used for downstream analysis.

Clinical annotations, such as survival time, vital status, age, and tumor stage, were extracted when available. Sample identifiers were harmonized across expression, clinical, and immune annotation files. Genes not detected in a given cohort were excluded from cohort-specific analyses.

### 4.2. Construction of the CMMS Score

A CMMS (CAF–Matrix–Metabolic Signature) was constructed by integrating three biologically defined modules: a contractile/myCAF activation module (C), a matrix-remodeling/ECM module (M), and a mitochondrial/metabolic module (Met). Representative genes were selected based on their highest connectivity in the co-expression network and established literature evidence as canonical markers. The contractile/myCAF module included ACTA2, TAGLN, MYL9, CNN1, TPM2, COL11A1, POSTN, PDGFRB, CXCL12, and IL6. The matrix-remodeling/ECM module included COL1A1, COL1A2, COL3A1, COL5A1, COL5A2, FN1, LOX, LOXL2, PLOD2, and SPARC. The mitochondrial/metabolic module included MFN1, MFN2, OPA1, DNM1L, RHOT1, TFAM, NDUFA4, SDHB, COX5A, and ATP5F1A (Table S3). Module-level activity scores were calculated for each sample, followed by z-score normalization within each cohort. The unweighted CMMS score was defined as the average of the three standardized module scores: CMMS = mean (C_z, M_z, Met_z). Patients were stratified into the CMMS-high and CMMS-low groups using the cohort-specific median CMMS score unless otherwise indicated.

### 4.3. LASSO-Weighted CMMS Model

To evaluate the prognostic contribution of individual CMMS genes, LASSO–Cox regression was performed using the 30 CMMS genes. All genes present in every dataset were encompassed by the analytical model. The analysis was conducted separately in TCGA-HGSOC, GSE32062, and GSE53963 to account for differences in cohort composition, platform characteristics, and expression distributions. The optimal penalty parameter was determined by cross-validation, and the lambda.min criterion was used to select the final model because the aim of this analysis was to assess the prognostic contribution of CMMS-related genes rather than to establish a highly parsimonious clinical prediction tool. Genes with non-zero coefficients at lambda.min were retained to calculate the cohort-specific LASSO-CMMS risk score as follows: risk score = Σ(coefficient_i × expression_i), where expression_i represents the normalized expression value of the corresponding gene. Patients in each cohort were divided into high-risk and low-risk groups according to the median risk score. The selected genes, coefficients, lambda.min values, number of non-zero genes, and exact risk-score formulas are provided in Table S4. Cross-validation curves and coefficient profiles are shown in Figure S1B–G. These LASSO-CMMS models were interpreted as cohort-specific prognostic summaries of CMMS-related genes and are not presented as a single transferable clinical classifier. Survival distributions were visualized by Kaplan–Meier curves, and the associations between risk groups and overall survival were quantified using Cox proportional hazards regression. The unweighted CMMS score was primarily used for biological association analyses, whereas the LASSO-weighted CMMS score was used for prognostic stratification.

### 4.4. Pathway and Functional Signature Scoring

Gene set activity scores were calculated using ssGSEA or module score-based approaches depending on the data type [36]. ECM, TGFβ, hypoxia, glycolysis, lactate-related, mTORC1, CD8 effector, and exhaustion signatures were evaluated using curated gene sets from hallmark pathways or published immune/stromal signatures.

In the bulk transcriptomic cohorts, gene-set activity was summarized for each individual sample. For single-cell RNA-seq analyses, module activities were quantified for individual cells with the AddModuleScore function. All signature scores were computed using genes detected in the corresponding dataset. When required, scores were standardized within each cohort before comparison or integration. A predefined exhaustion-related gene set, consisting of PDCD1, LAG3, TIGIT, HAVCR2, CTLA4, TOX, and CXCL13, was used to estimate exhaustion signature activity. The CD8 effector signature was calculated using cytotoxic T-cell effector genes, including GZMB, PRF1, GNLY, NKG7, and CTSW.

### 4.5. Immune Infiltration and Tumor Microenvironment Analysis

The tumor microenvironment composition was inferred using multiple complementary approaches. The overall immune and stromal content of individual tumors was evaluated using three parameters derived from the ESTIMATE algorithm: the immune score, stromal score, and tumor purity.

Bulk transcriptomic profiles were analyzed with ImmuCellAI and xCell to characterize immune cell infiltration. The inferred immune cell abundance for each sample was used for downstream comparisons between the CMMS-high and CMMS-low groups, defined by the cohort-specific median CMMS score. To compare immune cell infiltration levels across the predefined groups, the Wilcoxon rank-sum test was employed. Additionally, the association between the CMMS score and the abundance of various inferred immune cell populations was quantified using Spearman’s rank correlation analysis. Adjusted *p* values were obtained using the Benjamini–Hochberg method. Clustered correlation matrices were displayed using the corrplot package in R.

TIDE-associated metrics, including the TIDE score, dysfunction score, and exclusion score, were used to evaluate immune evasion phenotypes [29]. The association between CMMS and immune exclusion-associated phenotype or exhaustion was assessed using group comparisons, correlation analyses, and stratified analyses based on immune infiltration status.

Tumors were stratified into infiltration-high and infiltration-low subgroups using the median CD8 effector or immune infiltration score to evaluate the CMMS-associated effects across distinct immune contexts.

### 4.6. Mediation Analysis

Mediation analysis was performed to examine whether hypoxia mediated the association between CMMS and glycolysis. CMMS was treated as the exposure variable, the hypoxia score as the mediator, and the glycolysis score as the outcome variable. The mediation model quantified the hypoxia-mediated indirect effect, the residual direct effect, and the overall effect of CMMS on glycolysis. Statistical significance was assessed using nonparametric simulation or bootstrapping procedures. A significant indirect effect with attenuation of the direct effect was interpreted as evidence supporting a hypoxia-mediated relationship between CMMS and glycolytic remodeling.

### 4.7. Single-Cell RNA-Seq Data Processing

The single-cell RNA-seq datasets GSE154600 and GSE165897 were used to evaluate CMMS activity at the single-cell resolution. Raw or processed count matrices were analyzed using Seurat. For each dataset, low-quality cells were removed based on gene complexity, total UMI counts, and mitochondrial transcript percentage. Specifically, cells were retained if they met the following criteria: nFeature_RNA > 200, nFeature_RNA < 6000, nCount_RNA > 500, and percent.mt < 15%. Doublets were removed using original author-provided doublet filtering, and the final number of cells retained for each patient is summarized in Supplementary Tables S7 and S8.

After quality control, count matrices were normalized using Log Normalize, highly variable genes were identified, and the data were scaled before principal component analysis. Batch correction and dataset/sample integration were performed using RPCA-based Seurat integration, with 30 principal components used for downstream clustering and UMAP visualization. Cell clusters were identified using a graph-based clustering algorithm at a resolution of 0.8.

Canonical marker genes used for annotation included the following: EPCAM, KRT8, KRT18, and KRT19 for epithelial cells; COL1A1, COL1A2, DCN, LUM, PDGFRB, and ACTA2 for CAFs; PECAM1 and VWF for endothelial cells; CD3D, CD3E, NKG7, and CD8A for T/NK cells; LYZ and MS4A7 for myeloid cells; and MS4A1, CD79A, and JCHAIN for B/plasma cells (Table S9).

For single-cell statistical analyses, individual cells were not treated as independent biological replicates. Instead, cell-level scores and cell fractions were aggregated at the patient/sample level, and each patient/sample was used as the statistical unit for correlation or group-comparison analyses. Cell-level UMAPs, violin plots, and heatmaps were retained as descriptive visualizations.

### 4.8. CAF Subtype and Functional State Analysis

CAF subtypes were annotated based on known fibroblast markers and functional programs, including iCAF, myCAF, and TGFβ-CAF states. CMMS, ECM remodeling, TGFβ, hypoxia, and glycolysis scores were compared across CAF subtypes. For visualization, CAFs were classified as CMMS-high or CMMS-low using the dataset-specific median CMMS score among CAFs. Similarly, ECM-high CAFs were defined using the dataset-specific median ECM score among CAFs. Heatmaps and violin plots were used to visualize the functional heterogeneity among CAF subsets.

To reduce pseudo-replication from cell-level testing, statistical inference for key comparisons was performed at the patient/sample level. Cell-level UMAPs, violin plots, and heatmaps were used as descriptive visualizations, whereas quantitative comparisons were based on aggregated patient-level metrics. For each patient/sample, CAF-level metrics included the mean CAF CMMS score, mean ECM score, and the fraction of CMMS-high or ECM-high CAFs. Immune-related metrics included the CD8 T-cell fraction and mean CD8 exhaustion score within the same patient/sample. These values were correlated with the CD8 T-cell fraction and CD8 exhaustion score within the same sample. Only samples with sufficient numbers of CAFs and T cells were included in the sample-level correlation analyses.

### 4.9. Cell–Cell Communication Analysis

Intercellular communication networks were reconstructed with CellChat, focusing on ligand–receptor interactions between CAF subpopulations and T-cell subsets [10]. We classified CAFs into CMMS-high and CMMS-low subsets, while T cells were classified into exhausted and non-exhausted T-cell subsets according to exhaustion-associated marker expression. Communication probabilities were estimated for selected signaling pathways, with emphasis on matrix- and chemokine-related interactions, including COLLAGEN, FN1, LAMININ, CXCL, and CXCL12-CXCR4 signaling.

Outgoing signaling strength from CMMS-high and CMMS-low CAFs toward T-cell subsets was compared to identify CAF-derived communication programs potentially associated with T-cell exclusion or dysfunction.

### 4.10. Immunotherapy Cohort Analysis

The IMvigor210 anti-PD-L1-treated cohort was used as an exploratory immunotherapy-related validation dataset. Expression matrices and corresponding survival data were processed consistently with the aforementioned gene annotation and harmonization workflow. A LASSO-weighted CMMS risk score was calculated using available CMMS genes. A median-based cutoff was applied to classify patients into high- and low-risk groups. Survival outcomes were analyzed with Kaplan–Meier curves, and prognostic effects were estimated using Cox proportional hazards models. Because IMvigor210 is not an ovarian cancer cohort, this analysis was interpreted as exploratory evidence for the potential relevance of CMMS-associated stromal remodeling to immune checkpoint blockade response.

### 4.11. Overlap-Controlled and Leave-One-Module-Out Analyses

To evaluate whether ECM-related associations were driven by gene overlap with the CMMS definition, we performed overlap-controlled and leave-one-module-out sensitivity analyses. First, an external ECM-remodeling score was calculated using ECM-associated genes that were not included in the 30 CMMS genes. Second, three leave-one-module-out CMMS scores were generated by excluding the contractile/myCAF module, the matrix-remodeling/ECM module, or the mitochondrial/metabolic module. The resulting scores were defined as CMMS_no_C = mean (M_z, Met_z), CMMS_no_M = mean (C_z, Met_z), and CMMS_no_Met = mean (C_z, M_z). Spearman correlation analysis was then used to examine the associations between these alternative CMMS scores and the overlap-free ECM score. These analyses were used to distinguish built-in matrix–gene effects from broader stromal remodeling associations.

### 4.12. Cell Culture

The human fibroblast cell line MRC-5 was obtained from the Cell Bank of the Chinese Academy of Sciences (Shanghai, China). Primary human ovarian CAFs were isolated from ovarian cancer tissues collected at the Obstetrics and Gynecology Hospital of Fudan University. Primary human ovarian CAFs were isolated from 3 independent patient donors, and all in vitro experiments were conducted at passage P1–P3. The fibroblast phenotype was characterized by immunofluorescence staining for Vimentin. All human tissue specimens were obtained with institutional ethical approval and written informed consent from patients. Those above cells were cultured in an incubator with 5% CO_2_ and regularly sterilized at 37 °C. MRC-5 fibroblasts were stimulated by TGF-β1 (5 ng/mL) (PeproTech, Rocky Hill, NJ, USA) for 24 and 48 h in vitro. CAFs were subjected to the application of a TGFB1 neutralizing antibody (2 μg/mL) and were treated for 48 h.

### 4.13. Western Blot

MRC5 cells and CAFs were lysed in pre-chilled RIPA buffer. Following centrifugation, the supernatants were collected, and protein concentrations were quantified using a BCA assay. Protein samples were separated on 8–12% SDS-PAGE gels and transferred to 0.45 μm PVDF membranes. After blocking with 5% BSA, membranes were incubated with primary antibodies overnight at 4 °C and subsequently probed with HRP-conjugated secondary antibodies for 2 h at room temperature. Target proteins were visualized using an ECL chemiluminescence detection system. Band intensities were quantified using ImageJ v1.54f software. All primary and secondary antibodies used in this study are summarized in Table S1.

### 4.14. RT-qPCR

Total RNA was prepared with TRIzol reagent, reverse-transcribed using PrimeScript RT Master Mix (Takara Bio Inc., Kusatsu, Shiga, Japan; Cat. No. RR036A), and analyzed by qRT-PCR with SYBR Green II (Takara Bio Inc., Kusatsu, Shiga, Japan; Cat. No. RR820A). The primers utilized for qPCR are presented in the online Supplementary Table S2.

### 4.15. Immunofluorescent Staining

Paraffin-embedded tissues were deparaffinized, subjected to antigen retrieval, and blocked with 5% BSA. Sections were incubated overnight with the respective primary antibodies against Collagen I, α-SMA, CD8, or PDCD1, followed by HRP-conjugated or fluorescent secondary antibodies. IF staining employed DAPI for nuclei and confocal microscopy for imaging. For immunofluorescence staining, coverslip-cultured cells were sequentially fixed, permeabilized, blocked, and incubated with primary antibodies followed by Alexa Fluor-labeled secondary antibodies. Images were captured with a confocal microscope.

Multiplex immunofluorescence analysis was performed on HGSOC tissue sections from 80 patients. For each case, five representative non-necrotic ROIs were selected under identical imaging settings. Quantification was performed at the ROI level, and the mean value of five ROIs was used as the patient-level measurement. Therefore, each patient, rather than each ROI, was treated as the statistical unit (Table S10). CAF/ECM-rich status was defined based on the combined α-SMA and Collagen I signaling. For each region of interest, α-SMA^+^ and Collagen I^+^ areas were quantified as the percentage of total tissue area. The two values were z-score normalized and averaged to generate a CAF/ECM-rich score. Regions or samples with scores above the median were classified as CAF/ECM-high, whereas those below the median were classified as CAF/ECM-low.

### 4.16. CD8^+^ T-Cell Isolation, Coculture and Flow Cytometry

PBMCs were obtained and CD8^+^ T cells isolated by negative magnetic selection. Purified CD8^+^ T cells were activated using CD3/CD28 beads supplemented with IL-2 and expanded before being introduced into the co-culture system. In a transwell co-culture system, TGFβ1-pretreated MRC-5 fibroblasts were plated in the lower chambers of 24-well plates, while CD8^+^ T cells were seeded in the upper inserts. Activated CD8^+^ T cells were stained with viability and surface markers following standard protocols and were analyzed on a flow cytometer.

### 4.17. Statistical Analysis

Statistical analyses were performed in R. Comparisons between groups were performed using nonparametric tests, including the Wilcoxon rank-sum test for two-group comparisons and the Kruskal–Wallis test for multiple groups. Correlations between continuous variables were evaluated using Spearman’s method. Interaction effects were tested using Cox models containing the corresponding interaction terms. Two-sided *p* values < 0.05 were considered statistically significant.

## Data Availability

All datasets generated and analyzed herein are accessible via public databases, with corresponding repository names and accession codes provided within the manuscript.

## Supplementary Materials

The following supporting information can be downloaded at: https://www.mdpi.com/article/10.3390/ijms27136092/s1.

## Abbreviations

The following abbreviations are used in this manuscript:

CAF	Cancer-associated fibroblast
CMMS	CAF-associated mitochondrial metabolic and matrix-remodeling signature
HGSOC	High-grade serous ovarian carcinoma
ECM	Extracellular matrix
EOC	Epithelial ovarian cancer cell
ICB	Immune checkpoint blockade
