# Supplementary material for: A CAF-Associated Stromal Remodeling Signature Links Immune Exclusion to Exhaustion-Prone CD8+ T-Cell Dysfunction in High-Grade Serous Ovarian Cancer
Source: Int J Mol Sci. 2026 Jul 7;27(13):6092. doi: 10.3390/ijms27136092 (PMC13361325; doi:10.3390/ijms27136092)
Supplement: Supplementary file 1 [file ijms-27-06092-s001.zip › Figure legends.pdf]

Supplementary Figure S1. Expression landscape and LASSO-Cox modeling of CMMS genes.

(A) Boxplots showing the expression levels of the 30 CMMS genes in normal and tumor samples. Genes are ordered according to the three CMMS modules. (B) LASSO coefficient profile plot of candidate genes in the TCGA cohort. (C) Cross-validation partial likelihood deviance curve for LASSO-Cox regression in the TCGA cohort, with optimal lambda.min = 0.0911. (D) Density distribution of CMMS scores across GSE32062-HGSOC samples. (E) Cross-validation partial likelihood deviance curve for LASSO-Cox regression in the GSE32062 cohort, with optimal lambda.min = 0.0496.(F) LASSO coefficient profile plot of candidate genes in the GSE53963 cohort. (G) Cross-validation partial likelihood deviance curve for LASSO-Cox regression in the GSE53963 cohort, with optimal lambda.min = 0.0817. (H)Density distribution of CMMS scores across GSE32062-HGSOC samples. (I) Density distribution of CMMS scores across GSE53963-HGSOC samples. (J) Univariate Cox regression analysis for overall survival in patients with HGSOC. (K–M) Violin plots showing ECM signature scores between CMMS-high , CMMS-low groups in TCGA (K), GSE32062 (L), and GSE53963 (M). Intergroup Difference Comparisons Conducted via the Wilcoxon Rank-Sum Test. (N, O) Correlations between CMMS score and hallmark hypoxia scores across GSE32062 and GSE32062 datasets. Spearman correlation coefficients and P values are shown.

Supplementary Figure S2：Overlap-controlled and leave-one-module-out analyses of CMMS-associated ECM remodeling.

(A) Spearman correlations between the overlap-free ECM score and the original CMMS or leave-one-module-out CMMS scores in TCGA-HGSOC. (B) Heatmap summary of the corresponding Spearman correlation coefficients and P values.

(C, D) Scatter plots showing the correlations of the overlap-free ECM score with the original CMMS (C) and CMMS_no_M (D). Each dot represents one TCGA-HGSOC patient. (E) Boxplot comparing the overlap-free ECM score between CMMS_no_M-low and CMMS_no_M-high groups, stratified by the median CMMS_no_M score. P value was calculated using the Wilcoxon rank-sum test.

Supplementary Figure S3. Association of CMMS with tumor microenvironment scores and TGFβ activity across HGSOC cohorts.

(A) ImmuCellAI-based correlation matrix of CMMS and immune cell states in TCGA-HGSOC. (B-D) Comparison of TGFβ signature scores among CMMS-high, CMMS-low tumors in TCGA-HGSOC, GSE32062, and GSE53963 cohorts. (E) The GSE553963 cohort was used to compare CD8 effector signature scores between CMMS-high and CMMS-low tumors, and to analyze the Spearman correlation between CD8 effector and exhaustion signatures. (F) Scatter plot showing the relationship between nCount_RNA and nFeature_RNA across cells in GSE154600. (G) Violin plots summarizing single-cell quality-control metrics across samples. (H) Bar plot and summary table showing the number of cells retained before and after quality-control filtering for each sample.

Supplementary Figure S4. Cellular composition, differential markers, quality control and functional validation of CMMS in the GSE154600 and GSE165897 single-cell cohort.

(A) Cell-type composition across individual GSE154600 samples. (B) Summary of representative differential expression gen among CMMS-High, CMMS-Low CAFs. (C) Volcano plot of differential gene expression between CMMS-high, CMMS-low CAFs. (D) Distribution of nFeature_RNA, nCount_RNA, and mitochondrial transcript percentage in GSE165897. (E) UMAP visualization of refined cell-type annotations in GSE165897. (F) Association of patient-specific CAF CMMS with CD8 T cell

exhaustion, with corresponding CAF and CD8 T cell counts per patient in GSE165897. (G) Correlations between CMMS score and ECM or TGFβ scores in GSE165897. Spearman correlation coefficients and P values are shown. (H) Violin plots showing ECM, TGFβ, hypoxia, and glycolysis signature scores across refined cell types in GSE165897.(I) Dot plot showing expression of ligand–receptor-related genes across CMMS-high CAFs, CMMS-low CAFs, exhausted T cells, and non-exhausted T cells.

Supplementary Figure S5. Functional and communication features of CMMS-high CAFs in GSE165897.

(A) Heatmap showing representative marker genes differentially expressed between CMMS-high, CMMS-low CAFs. (B) GSEA plot showing the top positively enriched hallmark pathways in CMMS-high CAFs. (C) Ligand-receptor related gene expression in CAF_CMMS high/low and Tex/non-Tex T cells. Bubble size indicates the percentage of expressing cells, and color indicates average expression. (D) LASSO coefficient profile of the 29 CMMS-related genes based on Cox proportional hazards regression in IMvigor210.
