# Supplementary material for: A CAF-Associated Stromal Remodeling Signature Links Immune Exclusion to Exhaustion-Prone CD8+ T-Cell Dysfunction in High-Grade Serous Ovarian Cancer
Source: Int J Mol Sci. 2026 Jul 7;27(13):6092. doi: 10.3390/ijms27136092 (PMC13361325; doi:10.3390/ijms27136092)
Supplement: Supplementary file 1 [file ijms-27-06092-s001.zip › Supplementary Tables.pdf]

Table S1 Summary of antibodies used in the Western blot, Multiplex immunofluorescence and flow cytometry experiment

Antibody Name	Product Code	Dilution Ratio	Product Brand
Collagen Type I Rabbit Polyclonal Antibody	14695-1-AP	1：750	Proteintech
GAPDH Polyclonal antibody	10494-1-AP	1：500000	Proteintech
COL1A1 Rabbit Monoclonal Antibody	72026	1：1000	CST
Fibronectin/FN1 Rabbit Monoclonal Antibody	26836	1：1000	CST
alpha-Smooth Muscle Actin Rabbit Monoclonal Antibody	19245	1：1000	CST
Anti-Collagen I Rabbit pAb	GB115707	1:1000	Servicebio
Anti-alpha smooth muscle Actin Rabbit pAb	GB111364	1:500	Servicebio
Anti-PD1 Mouse mAb	GB12338	1；1500	Servicebio
Anti-CD8 alpha Rabbit pAb	GB115692	1：400	Servicebio
APC/Fire™ 750 anti-human CD45	304061	-	Biolegend
Fixable Viability Stain 510	564406	-	BD
Alexa Fluor 700 Mouse Anti-Human CD8(RPA-T8)	561026	-	BD
BV605 Mouse Anti-Human CD279 (PD-1) (EH12.1)	563245	-	BD

Table S2 Summary of PCR primers

Gene Names	Forward Primer（5'-3'）	Reverse Primer（5'-3'）
GAPDH	GACCCCTTCATTGACCTCAAC	CTTCTCCATGGTGGTGAAGA
FN-1	CAGTGGAGATGTTACGGACG	GCTGTCCTGAGTCAAATCGG
α-SMA	CTATGAGGGCTATGCCTTGCC	GCTCAGCAGTAGTAACGAAGGA
COL1A1	GAGAGCGAGGTGTTTGGAGA	ACCAGGGACCATTTGAGACC
POSTN	GACGCCATCAACACCGAGTT	CTTTGTCGTTGGTTAGCTGGT
CXCL12	ATTCTCAACACTCCAAACTGTGC	ACTTTAGCTTCGGGTCAATGC

Table S3 The complete gene list of CMMS score.

Contractile/myCAF	Matrix remodeling/ECM	Mito/metabolic
ACTA2, TAGLN, MYL9, CNN1, TPM2, COL11A1, POSTN, PDGFRB, CXCL12, IL6	COL1A1, COL1A2, COL3A1, COL5A1, COL5A2, FN1, LOX, LOXL2, PLOD2, SPARC	MFN1, MFN2, OPA1, DNM1L, RHOT1, TFAM, NDUFA4, SDHB, COX5A, ATP5F1A

Table S4 Parameters of cohort-specific LASSO-Cox models selected using the lambda.min criterion.

Cohort	Selected genes and Coefficients	Lambda.min value	Number of nonzero genes	Risk formula
TCGA	SDHB:0.4047<br>RHOT1:0.3395	0.0911	2	Risk Score = 0.4047 × SDHB + 0.3395 × RHOT1
GSE32062	PDGFRB: 0.0286<br>CXCL12: 0.0209<br>IL6: -0.0286<br>NDUFA4: 0.1958	0.0496207	4	Risk Score = 0.0286 × PDGFRB + 0.0209 × CXCL12 − 0.0286 × IL6 + 0.1958 × NDUFA4
GSE53963	PDGFRB: 0.0965<br>LOXL2: 0.5441<br>MFN2: 0.6868<br>RHOT1: 0.4311<br>NDUFA4: 0.3278	0.0817	5	Risk Score = 0.0965 × PDGFRB + 0.5441 × LOXL2 + 0.6868 × MFN2 + 0.4311 × RHOT1 + 0.3278 × NDUFA4
IMvigor210	IL6: -8.79e-05<br>TFAM: -2.93e-05<br>OPA1: -1.92e-05<br>COX5A: -9.68e-05<br>CNN1: -4.59e-06<br>MFN2: -9.32e-07<br>COL5A2: -2.34e-07	0.04683026	7	Risk Score = (-8.79×10^{-5}) IL6+(-2.93×10^{-5}) TFAM+(-1.92×10^{-5}) OPA1+(-9.68×10^{-5}) COX5A+(-4.59×10^{-6}) CNN1+(-9.32×10^{-7}) MFN2+(-2.34×10^{-7}) COL5A2

Footnote: LASSO-Cox models were fitted separately in each cohort using the lambda.min criterion. Risk scores were calculated as the sum of coefficient-weighted normalized gene expression values. These models were used for cohort-specific prognostic evaluation rather than for external validation of a single fixed classifier

Table S5: Availability of clinical covariates across cohorts.

Variable	**TCGA-HGSOC**	**GSE32062**	**GSE53963**
Age	Available	Available/Unavailable	Available/Unavailable
Stage	Available	Available/Unavailable	Available/Unavailable
Grade	Available	Available/Unavailable	Available/Unavailable
Residual disease	Incomplete/Unavailable	Unavailable	Unavailable
Treatment history	Incomplete/Unavailable	Unavailable	Unavailable
Platinum sensitivity	Unavailable/Incomplete	Unavailable	Unavailable
BRCA/HRD	Incomplete/Unavailable	Unavailable	Unavailable
Tumor purity	Estimated	Not available/Estimated	Not available/Estimated

Table S6. Univariable and multivariable Cox analyses of LASSO-CMMS risk in TCGA-HGSOC.

**Cohort**	**Variable**	Univariate			Multivariate		
		HR	95%CI	P value	HR	95%CI	P value
**TCGA-HGSOC**	Age	1.02	1.01-1.31	0.001	1.0253	1.0102–1.041	0.0010
	Stage (advance vs early)	2.32	0.74-7.30	0.149	2.5909	0.8233–8.154	0.1036
	CMMS group (high vs low)	1.30	1.02-1.32	0.037	--	--	--
	CMMS risk score	1.16	1.02-1.32	0.023	1.196	1.018-1.405	0.0293
	Tumor purity (per 0.1 increase)	0.99	0.89-1.10	0.804	0.9995	0.877-1.140	0.9994

Footnote: Multivariable Cox regression was performed in TCGA-HGSOC using available covariates, including age, FIGO stage, and estimated tumor purity. Tumor purity was modeled per 0.1 increase. CMMS group and CMMS risk score were not included in the same model to avoid collinearity. Comprehensive clinical adjustment was not performed in GSE32062 and GSE53963 because key clinical variables were unavailable or incompletely annotated.

Table S7. scRNA-seq QC summary

Dataset	Sample/patient	Before QC	After QC	Removed	Retention (%)
**GSE154600**	T59	16276	14792	1484	90.9
	T76	16678	15445	1233	92.6
	T77	8136	7627	509	93.7
	T89	5730	5297	433	92.4
	T90	4823	4422	401	91.7
**GSE165897**	EOC1005	-	3011	-	-
	EOC136	-	2501	-	-
	EOC153	-	1378	-	-
	EOC227	-	2156	-	-
	EOC3	-	3131	-	-
	EOC349	-	1480	-	-
	EOC372	-	687	-	-
	EOC443	-	2090	-	-
	EOC540	-	1886	-	-
	EOC733	-	1742	-	-
	EOC87	-	1484	-	-

Footnote: Before-QC cells not included in the uploaded GSE165897 files; this table summarizes post-QC cell numbers and patient-level metrics from the processed Seurat metadata/derived CSVs.

Table S8. Cell numbers per patient and annotation summary

Dataset	Sample/patient	EOC	Immune	Stromal	CAF cells	CD8T cells	CAF CMMS mean	CAF ECM mean	CD8 fraction of total (%)	Included analysis
**GSE165897**	EOC1005	427	2342	242	181	452	1.541	0.911	15.0%	Yes
	EOC136	205	1329	967	697	116	1.858	1.247	4.6%	Yes
	EOC153	328	641	409	440	40	0.930	0.645	2.9%	Yes
	EOC227	36	2089	31	28	189	0.260	0.212	8.8%	Yes
	EOC3	192	2588	351	375	149	1.378	0.814	4.8%	Yes
	EOC349	27	1096	357	92	396	1.236	0.874	26.8%	Yes
	EOC372	257	288	142	1121	34	0.554	0.527	4.9%	Yes
	EOC443	1054	612	424	363	27	2.212	1.293	1.3%	Yes
	EOC540	167	1537	182	235	102	0.935	0.748	5.4%	Yes
	EOC733	1550	149	43	17	1	1.025	0.712	0.1%	No
	EOC87	110	1079	295	311	156	1.838	1.033	10.5%	Yes

Footnote: CAF cells $\geqslant$20 and CD8 T cells $\geqslant$20. EOC733 is excluded from CAF-CD8 patient-level correlation because of insufficient CAF/CD8 cells.

Table S9. Annotation marker summary for GSE165897 single-cell analysis.

Major population	Observed subtype(s) in GSE165897	Canonical annotation markers
Epithelial tumor cells / EOC	EOC_C1, EOC_C10, EOC_C11, EOC_C12, EOC_C2, EOC_C3, EOC_C4, EOC_C5, EOC_C6, EOC_C7, EOC_C8, EOC_C9	EPCAM, KRT8, KRT18, KRT19
CAFs / stromal fibroblasts	CAF-1, CAF-2, CAF-3	COL1A1, COL1A2, DCN, LUM, PDGFRB, ACTA2
Mesothelial cells	Mesothelial	MSLN, KRT8, KRT18, KRT19
Endothelial cells	Endothelial	PECAM1, VWF, KDR
T/NK cells	T-cells, NK, ILC	CD3D, CD3E, CD8A, NKG7, GNLY
Myeloid cells	Macrophages, DC-1, DC-2, pDC, Mast-cells	LYZ, MS4A7, LST1, TYROBP, CD68
B/plasma cells	B-cells, Plasma-cells	MS4A1, CD79A, JCHAIN

Table S10. mIF cohort and quantification summary

Item	Description
Number of cases	HGSOC FFPE tissues, n = [80]
Inclusion criteria	pathologically confirmed HGSOC, available FFPE tissue, adequate tumor/stromal area
Exclusion criteria	severe necrosis, poor tissue preservation, staining failure, insufficient ROI
Markers	α-SMA, Collagen I, CD8, PDCD1, DAPI
ROI number	5 representative non-necrotic ROIs per case
Imaging	same microscope/platform, same magnification, identical exposure settings per channel
Quantification	α-SMA/Collagen I area fraction, CD8 density, $PDCD1^{+}CD8^{+}$ fraction or density
Group definition	CAF/ECM-high vs low by median α-SMA/Collagen I composite score
Statistical unit	patient-level mean of ROIs
