# Supplementary figures and images for: A CAF-Associated Stromal Remodeling Signature Links Immune Exclusion to Exhaustion-Prone CD8+ T-Cell Dysfunction in High-Grade Serous Ovarian Cancer
Source: Int J Mol Sci. 2026 Jul 7;27(13):6092. doi: 10.3390/ijms27136092 (PMC13361325; doi:10.3390/ijms27136092)

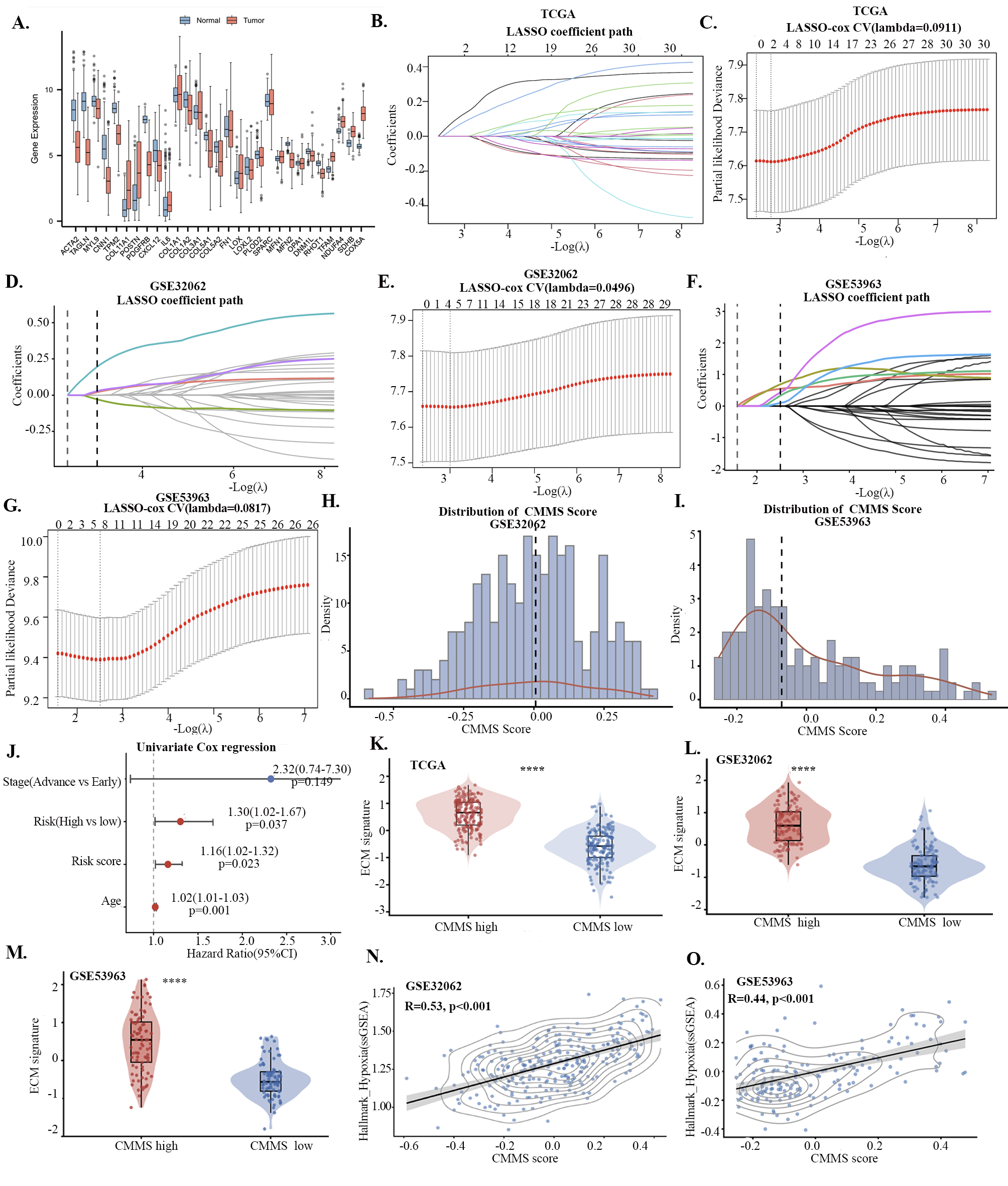

Supplement: Supplementary file 1 [file ijms-27-06092-s001.zip › supplemental figures/Figure S1.tif]

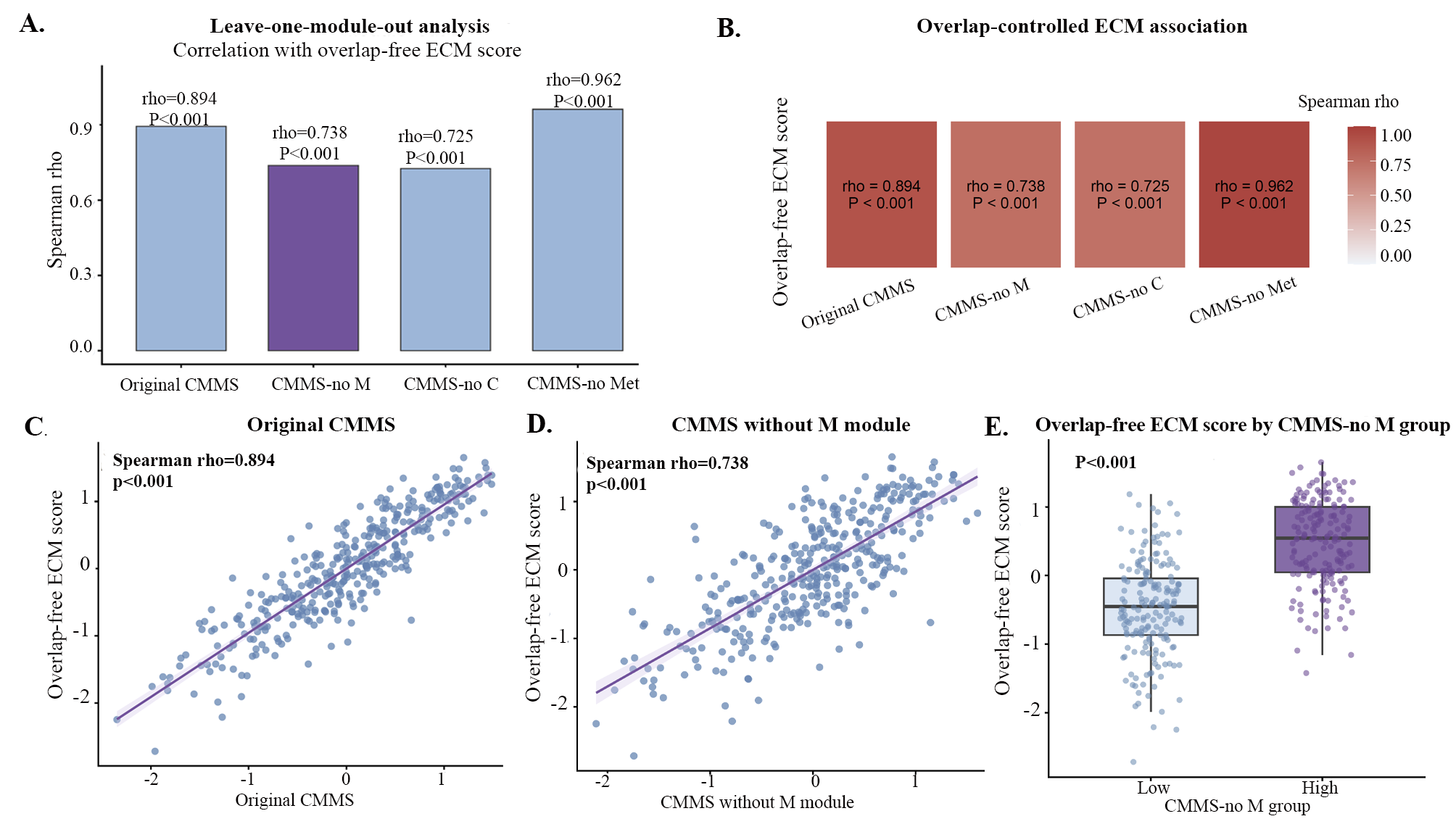

Supplement: Supplementary file 1 [file ijms-27-06092-s001.zip › supplemental figures/Figure S2.tif]

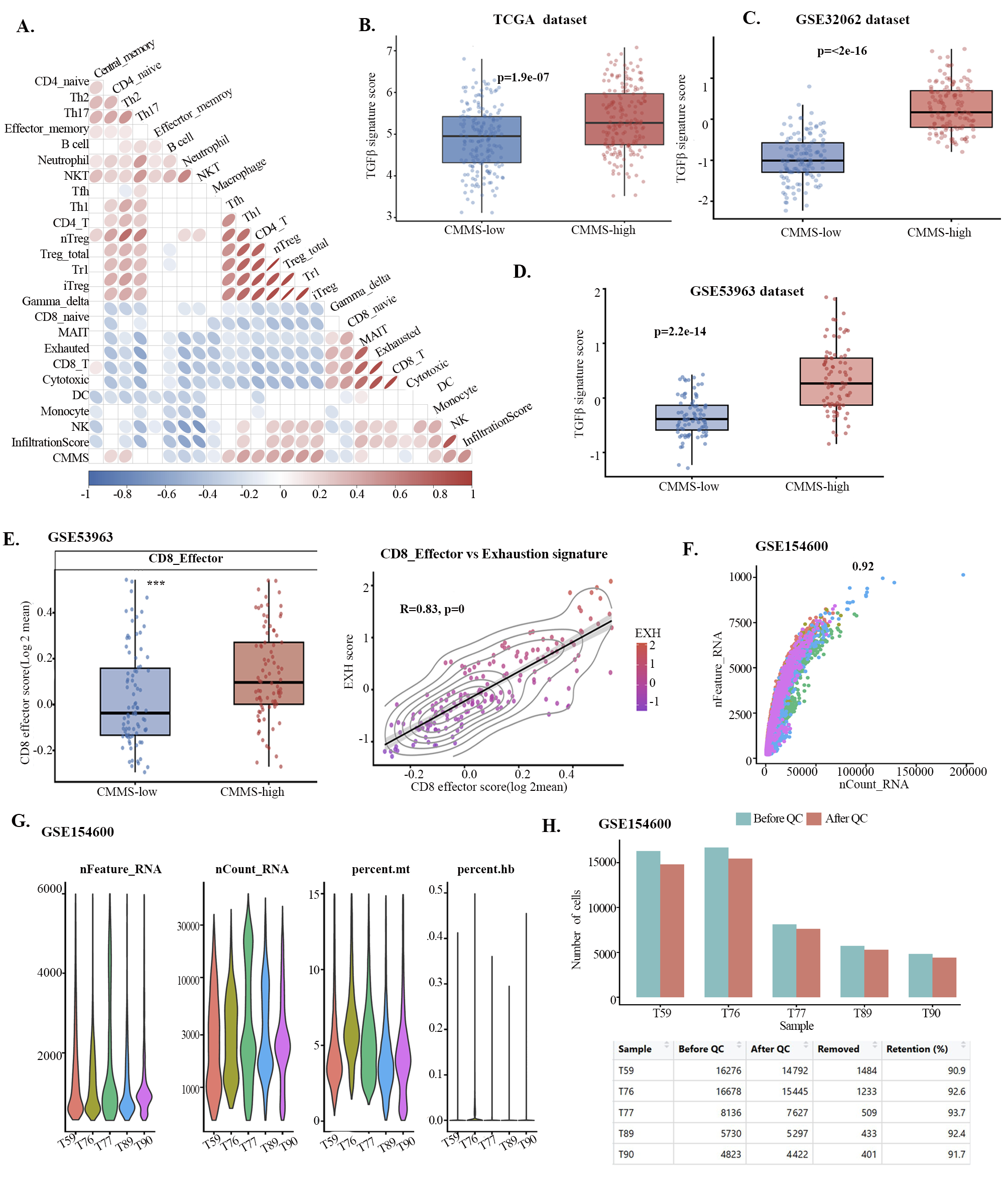

Supplement: Supplementary file 1 [file ijms-27-06092-s001.zip › supplemental figures/Figure S3.tif]

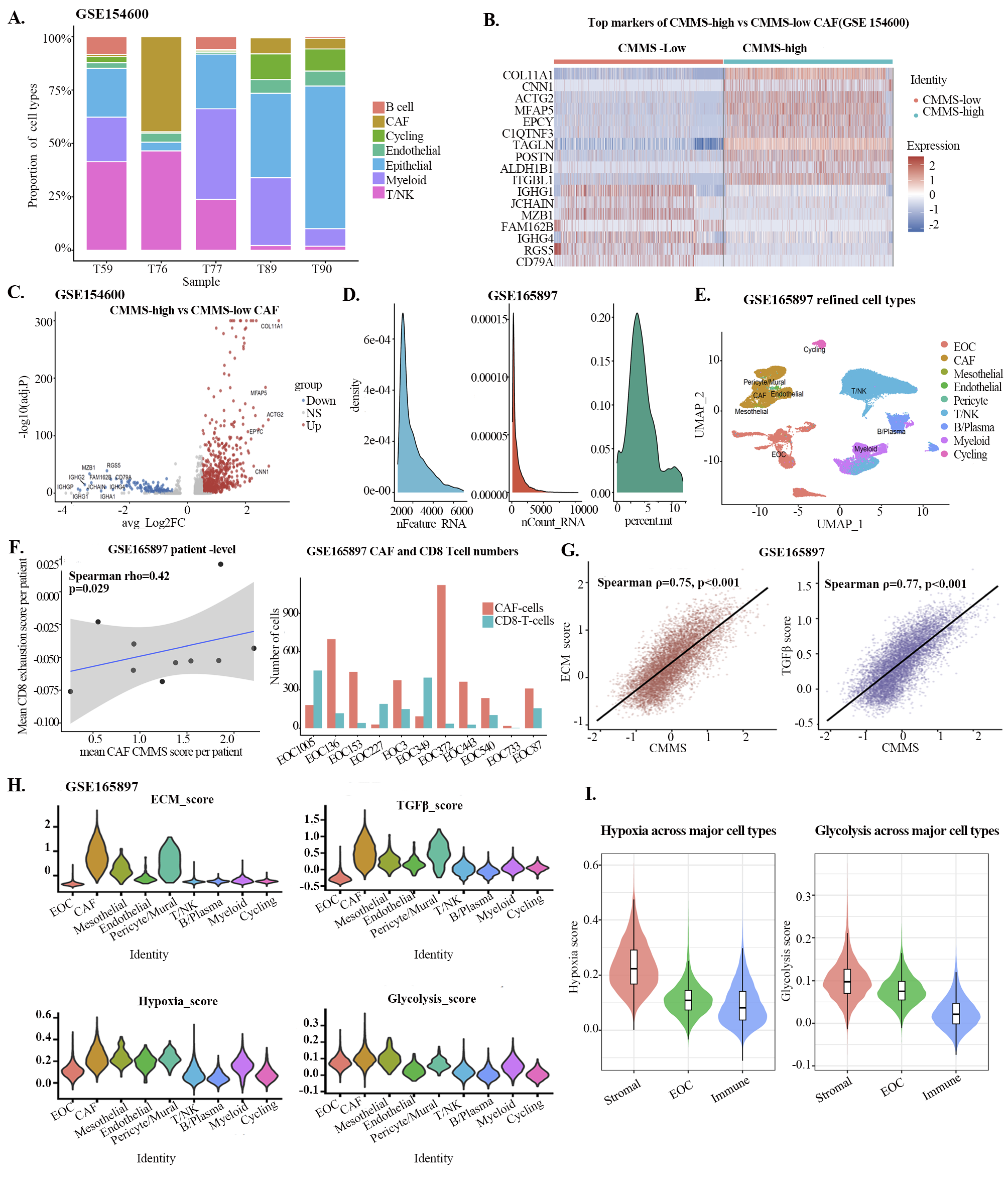

Supplement: Supplementary file 1 [file ijms-27-06092-s001.zip › supplemental figures/Figure S4.tif]

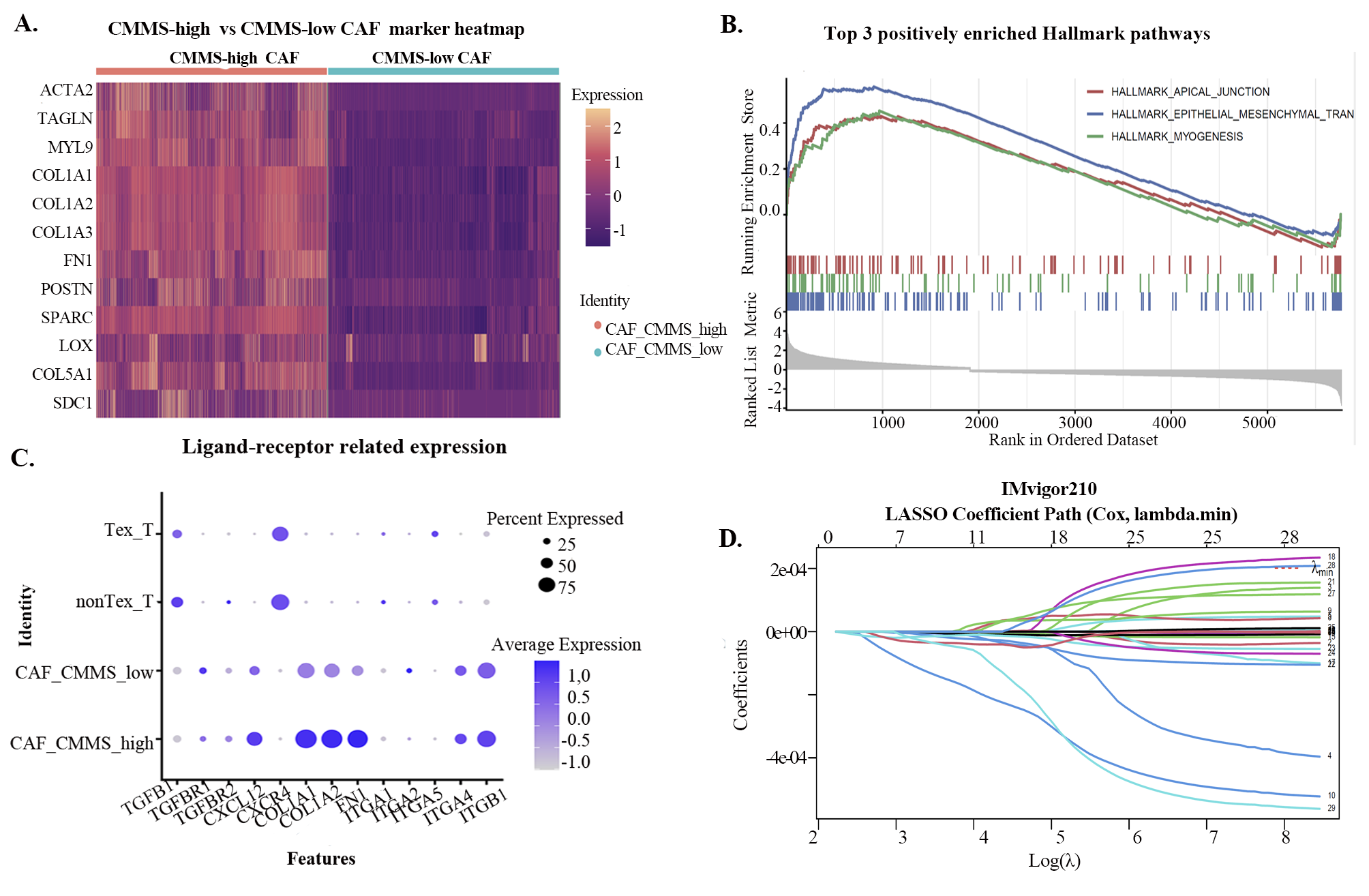

Supplement: Supplementary file 1 [file ijms-27-06092-s001.zip › supplemental figures/Figure S5.tif]
